# Discovery of a secreted *Bacteroides fragilis* mucinase that cleaves mucins with bis-T O-glycans through a carbohydrate binding module-dependent mechanism

**DOI:** 10.1080/19490976.2026.2644983

**Published:** 2026-03-19

**Authors:** Yoshiki Narimatsu, Cayetano Pleguezuelos-Manzano, Daniel Hornikx, Felix Goerdeler, Thapakorn Jaroentomeechai, Katia Flores, Sanae Narimatsu, Charelle Boot, Lars Hansen, Fabien Durbesson, Renaud Vincentelli, Laurie Comstock, Hans Clevers, Victor Taleb, Francisco Corzana, Bernard Henrissat, Henrik Clausen, Ramon Hurtado-Guerrero, Christian Büll

**Affiliations:** a Departments of Cellular and Molecular Medicine, Copenhagen Center for Glycomics & Center for Glycocalyx Research, Faculty of Health Sciences, University of Copenhagen, Copenhagen, Denmark; b GlycoDisplay ApS, Copenhagen, Denmark; c Hubrecht Institute, Royal Netherlands Academy of Arts and Sciences (KNAW) and UMC Utrecht, Utrecht, The Netherlands; d Department of Biomolecular Chemistry, Institute for Molecules and Materials, Radboud University, Nijmegen, The Netherlands; e Department of Biotechnology, Faculty of Science, Mahidol University, Bangkok, Thailand; f Department of Microbiology, Duchossois Family Institute, University of Chicago, Chicago, IL, USA; g Architecture et Fonction des Macromolecules Biologiques, Centre National de la Recherche Scientifique and Aix-Marseille University, Marseille, France; h The Institute for Biocomputation and Physics of Complex Systems (BIFI), Mariano Esquillor s/n, Zaragoza, Spain; i Departamento de Química and Instituto de Investigación en Química de la Universidad de La Rioja (IQUR), Logroño, Spain; j Department of Bioengineering and Biomedicine, Technical University of Denmark, Kongens Lyngby, Denmark; k Fundación ARAID, Zaragoza, Spain

**Keywords:** Mucinase, M60-like peptidases, mucins, O-glycans, microbiome, *bacteroides*

## Abstract

Degradation of mucins at the host–microbial mucus interphase involves glycosidases that release monosaccharides from O-glycans and mucinases that cleave the mucin protein backbone. Mucinases recognize and cleave peptide bonds at specific sequence motifs with varying O-glycan structures required and/or permissible. Mucinases that digest mucins with intact O-glycans can potentially destroy the protective mucus, while mucinases that only digest mucins with partially degraded O-glycans may serve at a later stage of nutrient sourcing from mucins. Here, we discovered nine CBM-bearing M60-like mucinases across gut commensals and opportunists, including a conserved *Bacteroides fragilis* mucinase denoted HC11. We also investigated the previously described *Bacteroides thetaiotaomicron* mucinase BT4244, which together delineates two functional classes with distinct preferences: BT4244 for bis-Tn (GalNAcα1-O-Ser/Thr) and HC11 for bis-T (Galβ1-3GalNAcα1-O-Ser/Thr) O-glycans. Both mucinases harbor carbohydrate-binding modules (CBM32) that bind their cognate O-glycan motifs and are required – together with the catalytic domains – for efficient cleavage of extended mucin domains, which is consistent with cooperative engagement, but are not required for the cleavage of short glycopeptides. We show *B. fragilis* strains secrete HC11 and degrade mucins only after the removal of sialic acids. Together, these findings expand the mucinase repertoire by nine enzymes spanning commensals and opportunists, demonstrate that CBM32 domains are essential for efficient cleavage of extended mucin substrates likely by promoting multivalent engagement and substrate positioning, and nominateidentify CBM–catalytic cooperation as a mechanism and intervention point for controlling mucus turnover and barrier integrity.

## Introduction

The mucus lining of mucosal body sites serves as protective barrier and is the interphase between the host and its resident microbiota.^1-3^ The mucus is primarily comprised of mucins which are large glycoproteins with a high density of O-glycans in O-glycodomains often found as tandem repeated (TR) sequence motifs.^4^^,^^5^ In the gut, the colonic epithelium and specialized Goblet cells continually produce mucins to maintain a thick mucus layer that forms the habitat for some members of the gut microbiome.^6^^,^^7^ Mucinophilic bacteria of the gut microbiota are highly adapted to this mucous environment and employ glycosidases and other enzymes to degrade mucins to source nutrients without destroying the protective mucus barrier.^8-11^ The result is the rapid turnover of the mucus and a gradient of mucins formed with increasingly eroded and simpler O-glycans from the underlying epithelium towards the lumen. The dense decoration of O-glycans in O-glycodomains makes mucins resistant to traditional peptidases, thus specialized O-glycopeptidases (mucinases) are required for degradation of the glycopeptide backbone of mucins.^12^^,^^13^ Most mucinases characterized so far cannot cleave nascent mucins with their fully elaborated O-glycans and only cleave mucins with eroded simple O-glycans such as the innermost Tn monosaccharide (GalNAcα1-O-Ser/Thr), or T disaccharide (Galβ1-3GalNAcα1-O-Ser/Thr) O-glycan structures.^14-19^ These mucinases produced mainly by the gut commensal microbiota may thus be considered beneficial or benign mucinases as they facilitate turnover of ageing mucins supporting mucus homeostasis. However, at least one class of mucinases represented by the StcE mucinase from the pathogenic enterohemorrhagic *Escherichia coli* (EHEC O157:H7) can efficiently cleave nascent mucins with elaborated O-glycans capped by sialic acids.^16^ Interestingly though, StcE does not appear to cleave mucins with core3 based O-glycans (GlcNAcβ1-3GalNAcα1-O-Ser/Thr),^20^ which commonly adorn the human mucin MUC2 that forms the mucus barrier in the intestine.^21^ These deleterious types of mucinases appear to be associated with pathogenic bacteria and are activated during infection,^22^^,^^23^ while mucinases that cleave only after trimming O-glycans are associated with gut symbionts. For example, the intestinal symbiont and mucin-degrader *Akkermansia muciniphila* secretes glycosidases that sequentially release monosaccharides from O-glycans and only produces the AM0627 mucinase that cleaves mucins with truncated O-glycans.^17^^,^^24^^,^^25^ It should be noted that some mucinases reported to degrade mucins with eroded O-glycans such as EatA and Pic produced by *E. coli* may also contribute to destruction and penetration of the mucus barrier,^26^^,^^27^ although further studies of the precise substrate specificities and functions of these enzymes are required.

Studies of the diversity of mucinases are revealing new families with differing structures and catalytic mechanisms.^28^ Most characterized mucinases classified in the MEROPS peptidase database belong to the M11, M26, M60, M72, and G9 families of metallopeptidases and StcE belongs to the M66 metallopeptidase family.^29^ Mucinases are multimodular proteins often carrying carbohydrate-binding modules (CBMs)^30^ or mucin-binding modules (MBMs),^31^ which are predicted to target mucinases to their substrates through cooperative binding.^19^ CBMs generally bind terminal (or rarely internal) motifs of oligosaccharides,^30^^,^^32^ while MBMs bind motifs formed by clusters of O-glycans.^31^ Direct evidence for the roles of CBMs/MBMs for mucinase function is still limited. We and others previously demonstrated that the StcE mucinase functions equally well in mucin degradation without its MBM (X409).^20^^,^^33^ Boraston and colleagues demonstrated that the *A. muciniphila* AMUC_1438 mucinase with a unique fold, functions like M60 mucinases, cleaving adjacent to single Tn O-glycans, with the CBM51 module enhancing enzymatic activity approximately 2-fold.^19^ However, the *A. muciniphila* AM0627 mucinase has a similar catalytic function but does not harbor CBMs.^17^ We recently characterized a di-glutamate *Bacillus cereus* K8 mucinase (denoted HC7) that utilizes its CBM5 to modulate and broaden its activity to include mucins with both Tn and T O-glycans.^28^

Here, we leveraged our recently published modular CBM walk strategy for mucinase discovery^28^ and a cell-based mucin array platform^20^ to identify nine previously uncharacterized, CBM-bearing M60-like mucinases distributed across gut commensals and opportunistic species, which segregate into two groups with distinct preferences for truncated bis-Tn or bis-T O-glycans. These mucinases required their CBM32 domains for cleavage of extended mucin substrates, but not for catalytic activity as they efficiently cleaved short glycopeptide substrates. Moreover, the glycan-binding specificities of the appended CBM32 modules follow the preferences for cleavage of mucins with Tn or T O-glycans. We found cooperative binding of the catalytic units and CBM32 modules to mucin substrates and employed site-directed mutagenesis and molecular dynamics simulations to demonstrate that the CBMs cooperate with the catalytic domains in cleaving extended mucin substrates. To our knowledge, these are the first mucinases shown to require their CBMs for efficient cleavage of extended substrates, establishing CBMs as indispensable for efficient processing of extended mucin substrates through substrate engagement and positioning.

## Results

### Novel M60-like mucinases

**Figure 1.**
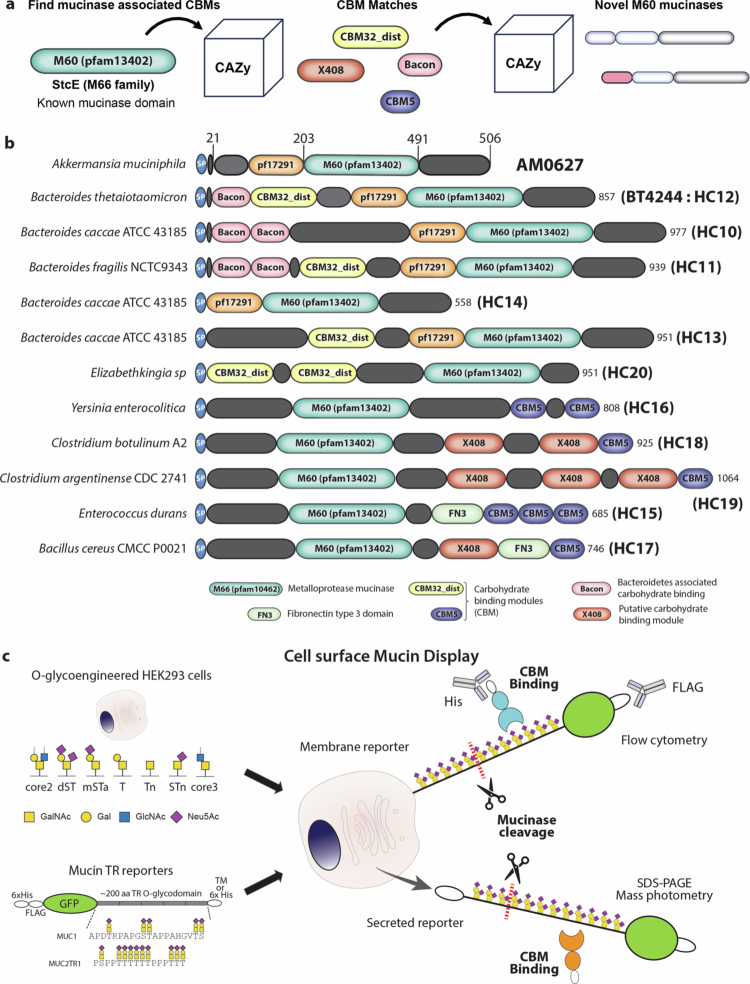
Discovery of M60-like mucinases. (a) Schematic overview of the modular walk strategy for the discovery of putative M60 domain-containing mucinases with carbohydrate binding modules (CBMs) using the CAZy database.^32^ (b) Schematic drawing of the modular domain architecture of identified putative M60-like mucinases from different bacteria, referred to as HC10 to HC20 in line with previously reported mucinases.^26^ Representation of the known mucinases AM0627 from *A. muciniphila* and BT4244 from *B. thetaiotaomicron* is shown. (c) Graphical depiction of glycoengineered HEK293 cells transfected with transmembrane or secreted mucin tandem repeat (TR) domain reporters carrying an *N*-terminal GFP and FLAG tags. HEK293 cells expressing membrane-bound mucin reporters are analyzed by flow cytometry to detect mucinase hydrolysis with high sensitivity by loss of the FLAG tag from the cell membrane or to quantify CBM binding to TR domains. Purified secreted mucin reporters with distinct O-glycosylation are used to detect mucinase activity by SDS-PAGE and western blot analysis as well as the CBM binding by mass photometry.

We previously reported preliminary results of a modular walk strategy for the discovery of novel mucinases by searching for CBMs with peptidase domains in the MEROPS database (Figure 1a).^28,34^ Here, we extended this strategy to the CAZy database,^35^ searching for potential mucinases among predicted CBM-containing proteins, and we identified ten mucinases (designated HC10-20) from different bacterial species ranging from symbionts colonizing the human gut (i.e., *B. fragilis*) to pathogenic *Clostridium botulinum* species that cause botulism. HC10-20 belong to the M60-like (Pfam 13402 containing) subfamily of zinc-metallopeptidases.^30^ This subfamily was previously noted to have CBMs,^30^ and the newly identified mucinases, referred to as HC10-20 for consistent numbering with previously identified mucinases from us,^28^ have different modules and CBMs (Figure 1b). Several members of the M60-like mucinase family were previously characterized in some detail, including *B. thetaiotaomicron* BT4244 bis-Tn O-glycan mucinase^24^^,^^30^^,^^36^ and *Akkermansia muciniphila* AM0627.^17^^,^^24^ The HC10-19 enzymes (full coding regions without signal peptide) and BT4244 mucinases were recombinantly expressed in *E. coli* and purified as shown in Supplementary Figure 1. The yield of HC20 was poor and this mucinase candidate was not further analyzed.

To study cleavage and binding properties of these mucinases, we employed our cell-based mucin array platform to display and produce glycoprotein reporters containing extended mucin O-glycodomains serving as natural mimics of human mucins.^20^ The cell-based mucin array platform provides mucin substrates of approximately 100‒200 amino acids derived from human mucin TRs decorated with custom-designed O-glycans produced in genetically glycoengineered HEK293 cells.^37^ These mucin reporters can be displayed on the surface of cells for analysis of mucin cleavage activity and binding properties by flow cytometry, and the reporters can be produced as secreted glycoproteins for analysis of cleavage and binding using biochemical assays (Figure 1c).

**Figure 2.**
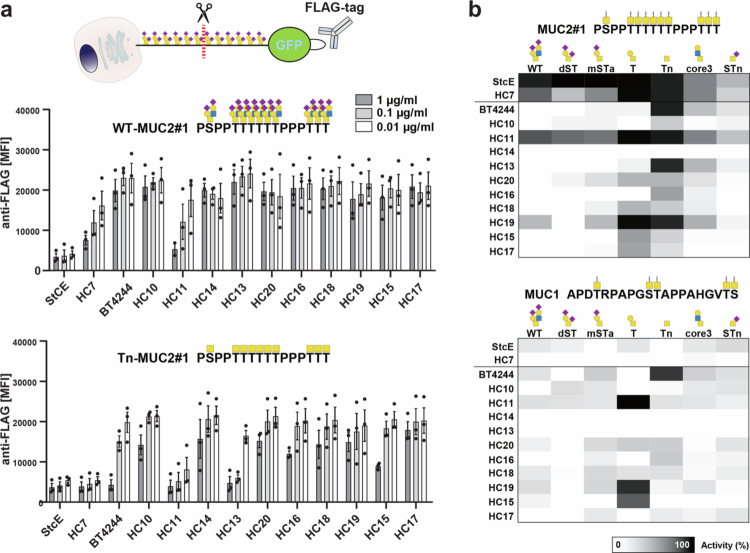
Mucinase activity screen using transmembrane mucin reporters reveals M60-derived mucinases and their glycan specificity. (a) Graphical depiction of glycoengineered HEK293 cells transfected with transmembrane mucin tandem repeat (TR) domain reporters carrying an *N*-terminal GFP and FLAG tag. Bar diagrams show activity of putative M60-derived mucinases on MUC2#1 reporters with wild type (WT) (upper diagram) and Tn (lower diagram) glycosylation. HEK293^WT^ cells and HEK293^KO *C1GALT1*^ cells displaying Tn (GalNAca1-O-Ser/Thr) were transiently transfected with transmembrane MUC2#1 reporters and treated with 0.01, 0.1, and 1 µg/ml of recombinant HC10-20, HC7, StcE, or BT4244. The bar diagrams show the average surface binding of APC-conjugated anti-FLAG antibody as mean fluorescence intensity (MFI) ±SEM of three independent experiments. (b) Substrate scope of HC10-20 for different O-glycan structures presented on MUC1 and MUC2. The transmembrane mucin reporters for MUC2#1 and MUC1 were expressed in HEK293 cells genetically engineered to produce O-glycans as indicated. Heat maps show representative activity of HC7, BT4244, and HC10-20 towards different glycoforms of MUC2#1 (top) and MUC1 (bottom) from two independent experiments. The activity was calculated based on the MFI of anti-FLAG tag binding normalized to that of the respective untreated HEK293 cell lines expressing MUC1 or MUC2 glycoforms.

We first demonstrated that most of the purified enzymes were active mucinases that cleave the purified reporter with dense clusters of the simple Tn O-glycans (Tn-MUC2#1), but not the corresponding reporter without O-glycans (naked-MUC2#1) (Supplementary Figure 2). The naked mucin reporter was produced in a CHO cell line with knockouts of all expressed polypeptide GalNAc-transferases (GALNTs) genes, resulting in reporters that lack O-glycans (not shown). Next, we used a HEK293 cell-based mucin array with cell surface display (membrane anchored) FLAG-tagged mucin reporters to monitor mucinase activity by loss of the extracellular *N*-terminal FLAG tag by flow cytometry (Figure 1c). These analyzes revealed that most of the HC10-19 mucinases predominantly cleaved mucins (MUC2#1) with simple Tn O-glycans, but not with complex O-glycans (mixture of sialylated core1/2 O-glycans) produced in HEK293 WT cells (Figure 2a). The known mucinases (StcE, BT4244, and HC7) were included as reference controls for evaluation of selectivity and efficiencies of the candidate mucinases. This is critical to discern substrate preferences in this assay because even a single cleavage event in the membrane-bound mucin reporters results in loss of the *N*-terminal GFP/FLAG tags rendering the cell-based mucinase assay very sensitive to enzyme dose and minor heterogeneity in the O-glycan make-up of the displayed reporters. Note, for example, that HC7 and HC11 appeared to exhibit cleavage of the cell surface reporter with complex O-glycans; however, this cleavage activity is relatively minor compared to the same reporter with Tn O-glycans (Figure 2a). We previously showed that HC7 efficiently cleaves only mucins with Tn and T O-glycans.^28^ Note that the apparent high cleavage of reporters displayed on cells with complex O-glycans is likely due to incomplete sialylation of the mucin reporters on cells resulting in single/low cleavage events and signal loss. An interesting observation was that the AM0627 mucinase without CBMs did not exhibit significant cleavage activity with membrane-bound mucin reporters, despite efficiently cleaving these as purified mucin reporters (Tn and T O-glycans) in solution (Supplementary Figure 3).

We proceeded to screen the identified mucinases against two HEK293 membrane-bound mucin reporters (MUC2#1 with high density and MUC1 with low density of O-glycans) (Figure 2b). Most of the mucinases preferentially cleave the Tn O-glycoforms similarly as the BT4244 mucinase; however, HC11 and HC19 preferentially cleave the T O-glycoform. These results suggest that the identified M60-like mucinases included two distinct classes of enzymes selectively cleaving mucins with bis-Tn or with bis-T O-glycans. The truncated Tn and T O-glycans are not found on nascent mucins with full elaborated O-glycans, but rather are the products of trimming by microbial glycosidases at the mucus host-microbial interphase.^9^^,^^10^

### Two classes of M60 mucinases with distinct bis-Tn/T O-glycoform preferences

**Figure 3.**
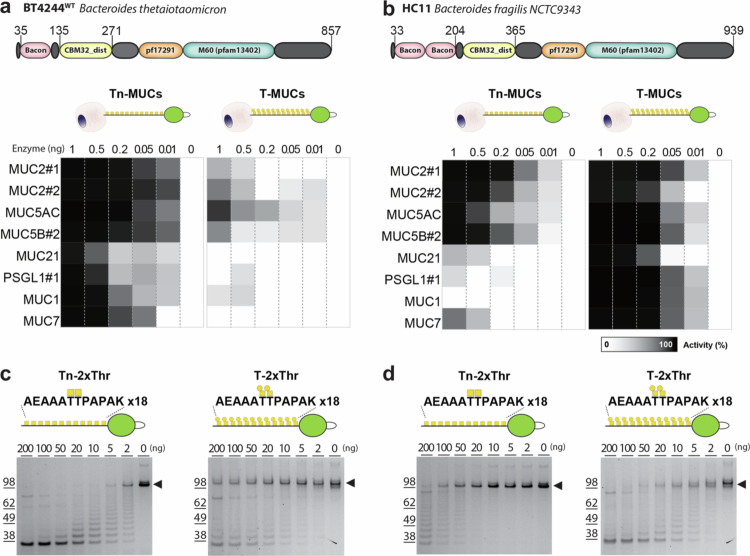
HC11 cleaves mucin domains with Tn O-glycans. (a,b) Schematic presentation of the modular organization of BT4244 (a) with an M60 protease family domain (pf17291, pfam13402) and a 1x BACON/CBM32_dist domain, and HC11 (b) containing an M60 protease family domain and a 2x BACON/CBM32_dist domain. Flow cytometry analysis of BT4244 and HC11 cleavage of FLAG-tagged transmembrane mucin(-like) TR reporters expressed in HEK293^KO *C1GALT1*^ (HEK293-Tn) or HEK293^KO *GCNT1, ST3GAL1/2, and ST6GANAC2/3/4*^ (HEK293-T) cells. The heat map shows representative activity calculated based on the MFI of anti-FLAG tag binding normalized to that of the respective untreated HEK293 cell lines expressing each mucin reporter. Full sequence of the mucin reporters are listed in Supplementary Figure 4. Data from two independent experiments are shown. (c,d) Digestion of soluble 2xThr reporters (for design see Supplementary Figure 5) with Tn or T O-glycans produced in HEK293-Tn and HEK293-T cells, respectively, by increasing concentrations of BT4244 and HC11. Purified mucin reporters (500 ng) were incubated with enzymes (0–200 ng dose titration) for 1 h at 37 °C and separated by Bis-Tris 4%–12% gels followed by staining with Krypton fluorescent protein stain. The gel image is representative of two independent experiments.

We selected BT4244 (HC12) and *B. fragilis* HC11 (Bf9343_2853) as representatives of each of the two classes of mucinases for further analysis. We analyzed their selectivity for different human mucin O-glycodomains using a panel of mucin reporters displayed on HEK293 cells producing either Tn or T (core1) O-glycans (Figure 3a and b, Supplementary Figure 4). This analysis confirmed that BT4244 preferentially cleaves mucins bearing Tn O-glycans and has only low activity with T O-glycosylated mucins. In contrast, HC11 efficiently cleaved all the mucins with T O-glycans but was less efficient at cleaving mucins with Tn O-glycans, particularly those with a lower density of O-glycans such as MUC1.

We also employed reporters that display identical repeats of simple O-glycan cluster motifs in a common random selected peptide sequence not designed from natural mucins (Supplementary Figure 5).^38^ Using these identical repeat reporters with a bis-Tn or bis-T dyad O-glycan cluster on a Thr-Thr sequon, we found that BT4244 exhibits high selectivity for Tn with minimal cleavage of the T O-glycan reporter (Figure 3c),^17^ while HC11 showed high selectivity for the T O-glycan reporter (Figure 3d). The *A. muciniphila* AM0627 mucinase in contrast showed similar activity for the bis-T and bis-Tn O-glycan reporters in solution (Supplementary Figure 3b), but as discussed above, AM0627 does not show activity with the same mucin reporters when presented on cells (Supplementary Figure 3a). Sequence analysis of these three proteins revealed a conserved tyrosine residue (Tyr723 in BT4244, Tyr817 in HC11, and Tyr470 in AM0627) that is absent in other mucinases, such as *Clostridium perfringens* ZmpB and ZmpC, which lack bis-O-glycan specificities (Supplementary Figure 3 and 8). This tyrosine is strategically positioned to engage O-glycans and plays a critical role in substrate recognition.^17^

### CBM32 is essential for activity with large mucin substrates, but not small glycopeptide substrates

**Figure 4.**
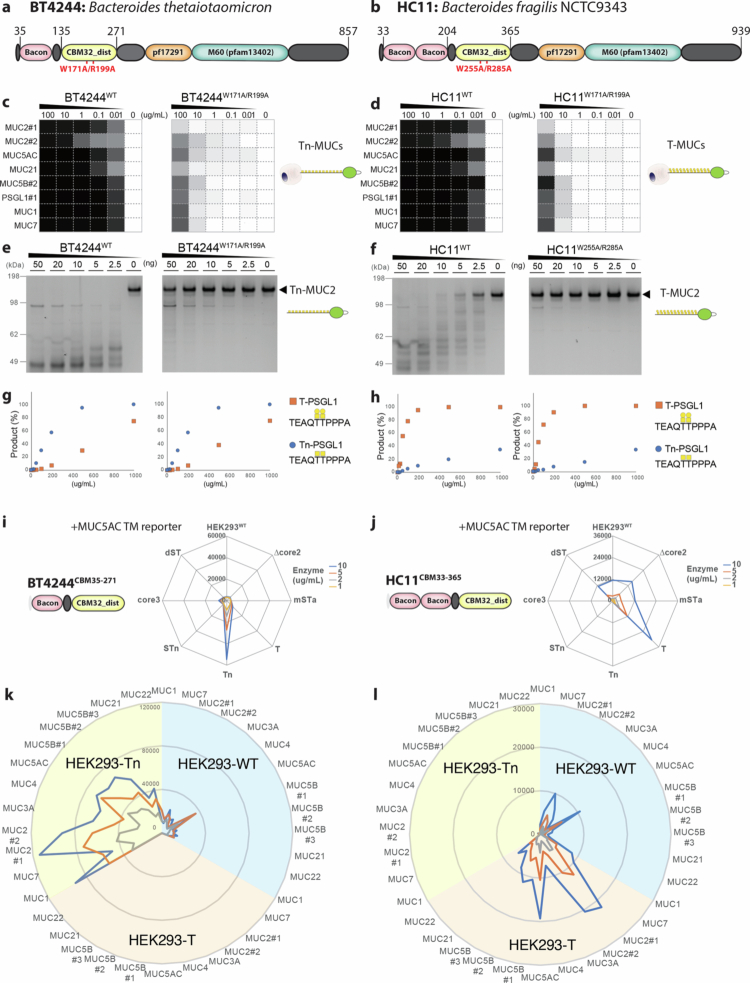
Carbohydrate-binding module of HC11 drives mucinase activity. (a,b) Schematic representation of BT4244 (a) and HC11 (b) expression constructs with CBM mutants targeting W171/R199A (BT4244^W171/R199A^) and W255/R285A (HC11^W255/R285A^), respectively. (c,d) Cleavage of membrane-bound mucin reporters stably expressed on glycoengineered HEK293 displaying Tn or T O-glycans by different concentrations (0–100 µg/mL) of wild-type (WT) parental and CBM mutant versions of BT4244 (c) and HC11 (d) was measured by flow cytometry. Heat maps show representative activity of WT and CBM mutants towards the cells expressing 8 different mucin reporters from two independent experiments. Activity was calculated based on the MFI of anti-FLAG tag binding normalized to that of the respective untreated HEK293 cell lines expressing each mucin reporter. (e,f) SDS-PAGE analysis of WT and CBM mutants cleavage for both BT4244 (e) and HC11 (f) with an isolated MUC2#1 reporter produced in glycoengineered HEK293 Tn or core1 cells. The purified reporters (0.5 µg) were incubated with enzymes (0–50 ng dose titration) for 1 h at 37 °C, separated by SDS-PAGE gels, and visualized using Krypton fluorescent protein stain. Representative gels of two independent experiments are shown. (g,h) Cleavage of synthetic PSGL1-like (TEAQTTPPPA) carrying bis Tn or T glycans by WT and CBM mutants of BT4244 (g) and HC11 (h) was analyzed using MALDI-TOF mass spectrometry. The cleavage activity was monitored by detecting the peak of the substrate and product by MALDI spectra and the % of the remaining substrate and product formation was semi-quantified and estimated. (i-l) Flow cytometry analysis of 1xBACON/CBM32_dist (BT4244^CBM35-271^) and 2xBACON/CBM32_dist (HC11^CBM33-365^) CBM domain binding to O-glycoforms of membrane-bound mucin reporters expressed in glycoengineered HEK293 cells. Radar plots show binding of different concentrations of BT4244^CBM35-271^ and HC11^CBM33-365^ to the MUC5AC reporter stably expressed on glycoengineered HEK293 cells displaying different O-glycoforms (i, j) and different membrane-bound mucin reporters transiently expressed on HEK293-WT (blue), HEK293-T (orange), or HEK293-Tn (yellow) cells (k,l). Binding was quantified by flow cytometry, and the data are shown as representative MFI values of two independent experiments.

Hirt and colleagues originally found that the subfamily of M60-like (Pfam 13402 containing) zinc-metallopeptidases contain CBMs.^30^ The AM0627 mucinase does not contain CBMs; however, both BT4244 and HC11 contain bacteroides-associated carbohydrate-binding often *N*-terminal (BACON) and CBM32 domains (Figure 4). To study the function of the BACON/CBM32 domain, we first expressed BT4244 and HC11 without these (BT4244^∆CBM^ and HC11^ΔCBM^) and demonstrated near complete loss of activity towards membrane-bound and soluble mucin reporters (Supplementary Figure 6). We then produced BT4244 and HC11 with CBM32 inactivating mutations (W171A/R199A for BT4244 and W255A/R285A for HC11) predicted from AlphaFold2 structures to abrogate glycan-binding, and confirmed that both mucinases lost near complete cleavage activity of mucin reporters displayed on cells (Figure 4c and d) and purified reporters in solution (Figure 4e and f). Remarkably though, the catalytic activities of both BT4244 and HC11 mucinases remained unaltered when tested with short glycopeptides (10-mers) carrying a single bis-Tn or bis-T O-glycan dyad, while still retaining their preferences for Tn and T O-glycan substrates, respectively (Figure 4g and h). This finding shows that the catalytic units are active but require the cooperative binding of the glycan-binding CBMs to access and digest extended O-glycodomains of mucin substrates. Thus, we propose that the CBM32 modules enhance binding and enforce a productive substrate geometry.

### CBM32 variants of BT4244 and HC11 differ in O-glycan binding properties

Next, we examined the glycan-binding properties of the isolated BACON/CBM32 domains of BT4244 and HC11. We were unable to express and produce the individual CBM32 domains alone (data not shown). However, the combined BACON/CBM32 domains were expressed and these exhibited striking differences in binding to the Tn and T O-glycans displayed on the membrane-bound MUC5AC mucin reporter on glycoengineered HEK293 cells (Figure 4i and j). The BT4244 BACON/CBM32 domain specifically bound to Tn, whereas the HC11 BACON_2x_/CBM32 specifically recognized T O-glycans, mirroring the mucin substrate cleavage specificities of their respective mucinases. We further tested binding to glycoengineered HEK293 cells displaying a panel of mucin reporters with different O-glycans structures by flow cytometry (Figure 4k and l), which demonstrated that these BACON/CBM32 variants bound T or Tn O-glycans largely independent of the contextual presentation of O-glycans in clusters and patterns found on mucins. We were unable to directly confirm that the glycan-binding properties reside in the CBM32 domains; however, this family of CBMs contains numerous validated variants with well-established Gal/GalNAc binding properties.^39^ No carbohydrate-binding function has so far been demonstrated for BACON domains, and this domain has been suggested to act as a spacer or mobility element for catalytic domains.^40^

### Coordinate glycan-binding of CBM32 and catalytic mucinase domains

**Figure 5.**
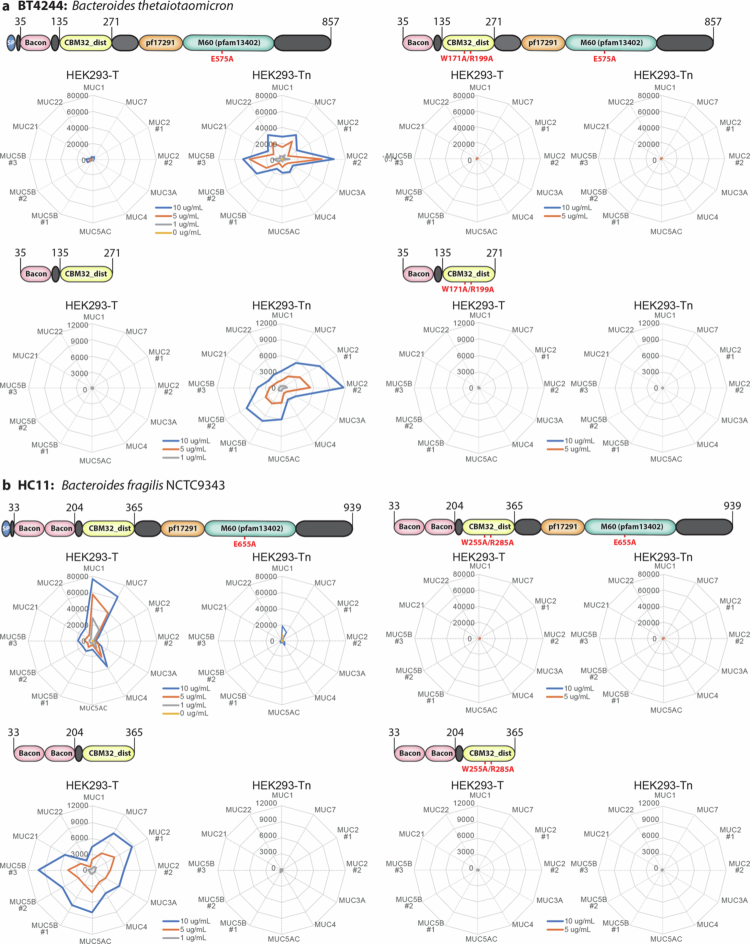
Dissecting cooperative binding of the CBM and catalytic unit of mucinases. (a) Flow cytometry analysis of mucin-binding properties of BT4244 employing the full-length BT4244^E575A^ catalytically inactivated mutant, its CBM32 inactivated double mutant (BT4244^E575A;W171A/R199A^), and the independent BACON-CBM32 WT and mutant (W171A/R199A) module. (b) Flow cytometry analysis of HC11 employing the full-length HC11^E655A^ catalytically inactivated mutant, its CBM32 inactivated double mutant (HC11^E655A;W255A/R285A^), and the independent BACON-CBM32 WT and mutant (W255A/R285A) modules. Twelve membrane-bound mucin reporters were transiently expressed in HEK293-T or HEK293-Tn cells. Radar plots show presentative binding data as MFI values of two independent experiments.

We next explored the potential cooperative binding of the CBMs and catalytic domains. The Bertozzi group previously used a catalytically inactivated StcE mucinase (StcE^E447D^) to evaluate the glycan- and mucin-binding properties of this enzyme.^16^ It is now clear that the catalytic domain is directed to disialyl-T (dST) O-glycans,^33^ while the X409 MBM is dispensable for the catalytic activity of the StcE mucinase but mediates its unique mucin-binding properties.^20^^,^^31^ The BT4244 and HC11 mucinases represent excellent models to study cooperative binding as we found that the CBM32 domains are required for mucinase activity. We therefore tested the binding properties of these enzymes with and without inactivating mutations in the catalytic domains (BT4244: E575A, HC11: E3333655A) and CBM32 domains (BT4244: W171A/R199A, HC11: W255A/R285A) employing the cell-based mucin array (Figure 5). The isolated proteins with and without these mutations were included for comparison, confirming that the mutants were inactive. The double-inactivated mutants of both mucinases (BT4244^E575A;W171A/R199A^ and HC11^E655A;W255A/R285A^) showed no binding to mucins, while the catalytic inactive mutants (BT4244^E575A^ and HC11^E655A^) with intact CBM32 showed binding to Tn and T mucins, respectively, as expected. Surprisingly, the catalytic mutants exhibited markedly enhanced (5–10-fold) binding in comparison to the binding observed with the isolated BACON/CBM32 domains (Figure 5). Moreover, this binding enhancement appeared to be selective for mucins with high and low densities of O-glycans, respectively. This effect was less obvious for BT4244^E575A^, where binding to the high-density mucins MUC2 and MUC5B with Tn O-glycans appeared selectively enhanced (Figure 5a), but HC11^E655A^ exhibited highly selective binding to the low-density mucins MUC1 and MUC7 with T O-glycans (Figure 5b). Interestingly, MUC1 and MUC7 are characterized by having bis O-glycans. We further confirmed the specific binding of BT4244^E575A^ to high-density Tn mucin reporters (i.e., MUC5AC) and HC11^E655A^ binding to low-density mucin reporters (i.e., MUC1) by single molecule mass photometry analysis (Supplementary Figure 7).

These results suggest that cooperative binding of the catalytic units and CBM32 of the BT4244 and HC11 mucinases is critical for both binding to and cleavage of mucin substrates, but interestingly not for cleavage of short glycopeptides (Figure 4g and h). This may, at least partly, explain why the AM0627 mucinase without CBMs exhibits negligible cleavage of mucin reporters displayed on the cell membrane while cleaving isolated mucin reporters in solution (Supplementary Figure 3) and short glycopeptide substrates.^17^

### Molecular dynamics (MD) simulations suggest that CBM32 domains contribute to substrate positioning

To investigate the role of CBM32 in catalysis, we initially sought to resolve the structural basis of substrate engagement through cocrystallization of HC11 and BT4244 mucinases. We used synthetic 16-mer Tn and T O-glycopeptides designed from the MUC1 TR (APGS*T*APPAHGVT*S*AP, * indicating Tn/T O-glycans) with two bis O-glycan dyad spaced seven residues apart, but despite optimization efforts complexes did not yield crystals suitable for X-ray diffraction. We therefore used AlphaFold3 simulations of the full sequences of HC11 and BT4244 which suggested that the CBM32 domains were positioned near the catalytic domains (Supplementary Figure 8). We manually docked the 16-mer MUC1 glycopeptides into the AlphaFold models by orienting the *N*-terminal bis O-glycan into the CBM32 binding pocket and the C-terminal into the catalytic site, following the binding mode reported for AM0627 mucinase (Supplementary Figure 9).^17^ We performed 300-ns restrained MD simulations to probe how different glycoforms might be accommodated across the CBMs and catalytic domains while preserving a plausible initial binding pose. The trajectories highlight glycoform-dependent interaction patterns, which we use as a qualitative framework to support the biochemical data. For HC11 the bis-T glycopeptide was more stably accommodated than the bis-Tn glycopeptide, maintaining persistent interactions across both the CBM32 and catalytic domains throughout the simulation. The GalNAc moiety of the T O-glycans engaged strongly with CBM32 residues Trp255 (via CH/π interaction) and Arg285 (via hydrogen bonding), likely stabilized by the additional galactose residue in the T O-glycan. At the catalytic site, the Gal on Thr13 and the GalNAc on Ser14 in the C-terminal bis T O-glycans consistently interacted with Tyr817 and Tyr620, indicating that the bis-T glycopeptide adopts a more competent binding mode for cleavage. For BT4244, positioning glycopeptides similarly revealed that the *N*-terminal bis-Tn glycopeptide exhibited better stabilization through both CH/π and hydrogen-bond interactions with the CBM32 domain. The BT4244 catalytic cleft favored Tn O-glycans forming slightly stronger and more persistent interactions across both domains. Comparatively, the simulations indicate that CH/π stacking between GalNAc and a conserved tryptophan is preserved in both enzymes and glycoforms, with minor variations, whereas the hydrogen bond involving the sugar carbonyl and arginine differs by glycoform, tighter for Tn in BT4244 than in HC11 and comparable for core 1 in both enzymes. Distance analyzes further support these observations, confirming the persistence and strength of glycopeptide–residue contacts (Supplementary Figure 9). Together, these simulations are consistent with a model in which the CBM32 domains on HC11 and BT4244 help position extended glycopeptide substrates relative to the catalytic cleft and may help explain the observed glycoform preferences together with catalytic-site determinants.

### HC11 is a major T-mucinase of *B. fragilis* able to cleave native human gut organoid-derived mucins

**Figure 6.**
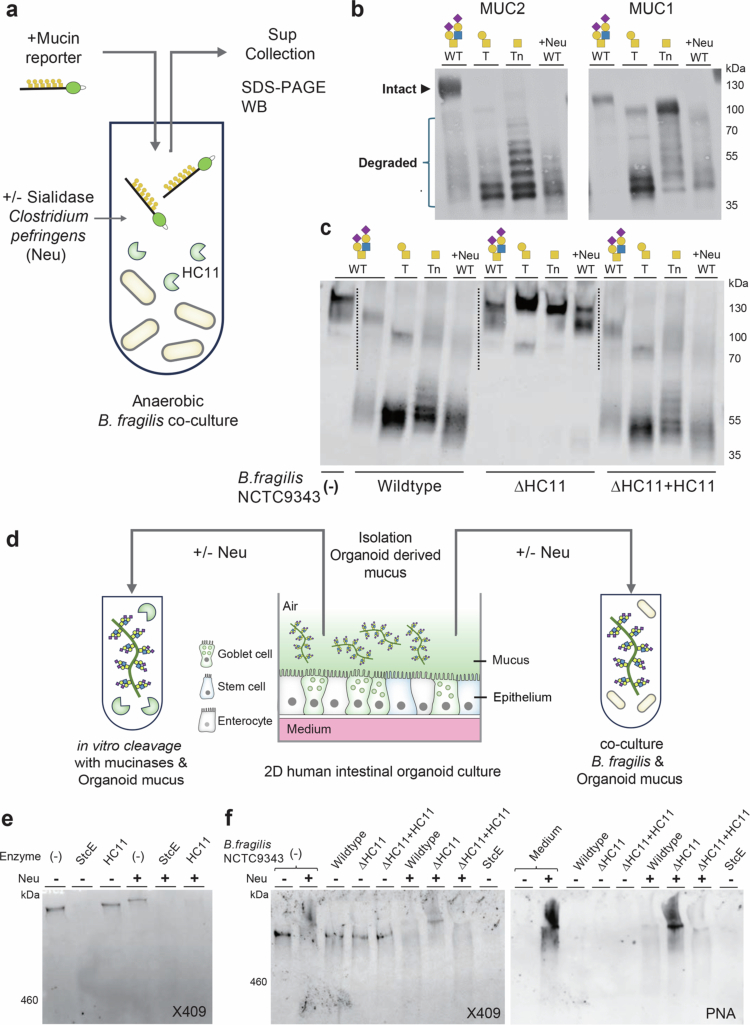
HC11 is a major secreted mucinase of *B. fragilis*. (a) Schematic overview of the assay design for mucinase activity of *B. fragilis* in anaerobic culture with mucin reporters. (b,c) SDS-PAGE Western blot analysis (anti-FLAG) showing degradation of mucin reporter glycoforms by the *B. fragilis* NCTC 9343 wild-type strain (b) and by an HC11 knock-out strain (ΔHC11) and an HC11 rescued strain (ΔHC11 + HC11) (c). Mucin reporters were incubated for 24 h, and +Neu indicates that the recombinant *C. perfringens* sialidase (Neu) was added to the cultures. Representative blots from three independent experiments are shown. (d) Schematic overview of the assay design for HC11mucinase activity using mucus produced by human adult stem cell-derived intestinal organoids grown in 2D on transwell culture inserts with an air (apical)/liquid (basal) interface. (e) Western blot analysis of the digestion of secreted organoid mucus with the mucin-binding probe X409-GFP MBM. Mucus was treated with recombinant HC11 or StcE mucinases with or without *C. perfringens* sialidase, as indicated. (f) Western blot analysis of the digestion of secreted organoid mucus in culture assays with *B. fragilis* strains, as indicated. Anerobic culture was performed for 24 h with or without *C. perfringens* sialidase. Recombinant StcE mucinase included as control and X409-GFP MBM (left) was used to detect mucins and the PNA lectin (right) to monitor the release of sialic acids and the exposure of mucins with T O-glycans. Blots are representative of two independent experiments.

HC11 is encoded by a conserved gene of *B. fragilis*, a member of the human gut microbiota (Figure 1b), which prompted us to explore the mucin-degrading properties of *B. fragilis*. We grew *B. fragilis* strain NCTC9343 under anaerobic conditions in the presence of mucin reporters with different O-glycans (Figure 6a). We analyzed the degradation of these mucin reporters after 24 h culture from the culture supernatant by SDS-PAGE Western blotting and found efficient degradation of T-MUC2 and T-MUC1 reporters and only limited degradation of the same reporters with Tn-O-glycans. Essentially, no degradation was observed with reporters containing complex O-glycans (WT with sialylated core1/2 O-glycans) (Figure 6b). This result corroborates the identified bis-T mucin substrate specificity of HC11. Since the removal of sialic acids from WT mucin reporters with complex O-glycans will expose bis-T O-glycans, we then tested digestion in the presence of an exogenous sialidase from *C. perfringens*, which resulted in efficient degradation of the WT mucin reporters (Figure 6b). Notably, when a distinct gut Bacteroides species, *Parabacteroides distasonis* strain DSM20701 lacking HC11 mucinase, was cultured with these mucin reporters, no degradation was observed (Supplementary Figure 10a). To further confirm that the observed mucin-degrading activity of *B. fragilis* NCTC9343 is mediated by HC11, we generated a deletion mutant of *B. fragilis*^ΔHC11^ NCTC 9343 (deletion of BF9343_2853) and demonstrated that this mutant completely lost the ability to degrade the mucin reporters, while restoration of this gene to the mutant strain fully restored mucin-degradation (Figure 6c). The same results were obtained by mutation and complementation of the *B. fragilis* enterotoxin (BFT) containing strain *B. fragilis* 86-5443-2-2 (Supplementary Figure 11). Mucin-degrading activity with selectivity for T-mucin reporters was also observed in conditioned medium derived from overnight *B. fragilis* cultures suggesting that the HC11 mucinase is secreted (Supplementary Figure 10b). These data show that HC11 is responsible for mucin degradation in *B. fragilis*, a gut symbiont that unlike *B. thetaiotaomicron*, utilizes only a few plant polysaccharides and tends to specialize in host mucin O-glycan utilization.^41^

Finally, we explored HC11-mediated degradation of native mucus isolated from human adult stem cell-derived intestinal organoids differentiated in a 2D transwell system (Figure 6d). The isolated mucus was either mixed with recombinantly expressed mucinases or with live *B. fragilis* NCTC9343 bacteria. We employed the X409 MBM that recognize all glycoforms of MUC2 to probe degradation of the native mucus by Western blotting (Figure 6e).^20^^,^^31^ X409 MBM staining detected a high molecular weight (HMW) band (>460 kDa marker) likely representing MUC2 in the mucus isolated from the intestinal organoids (Figure 6e), and the X409 MBM reactivity was fully eliminated by the StcE mucinase that efficiently cleaves MUC2 with complex sialylated O-glycans. This HMW band was insensitive to direct treatment with HC11, but fully eliminated by HC11 in the presence of an exogenous sialidase (Figure 6e), demonstrating that the native mucins in the mucus of human organoids are well glycosylated and resistant to HC11 degradation unless desialylated. We then cultured the organoid-derived mucus together with the *B. fragilis* NCTC9343 wild-type bacterium and HC11 mutant strains, with and without addition of an exogenous sialidase, which recapitulated that the *B. fragilis* HC11 mucinase cleaved mucins only following the removal of sialic acids (Figure 6f). We further confirmed that the X409 MBM-reactive HMW mucin band carries ST O-glycans as it was labeled with PNA lectin (binds T O-glycans) only after sialidase treatment (Figure 6f). These results demonstrate that the *B. fragilis* HC11 mucinase does not digest nascent mucins with elaborate O-glycans, but serves in mucin degradation only after microbial erosion of O-glycans by exoglycosidases.

## Discussion

**Figure 7.**
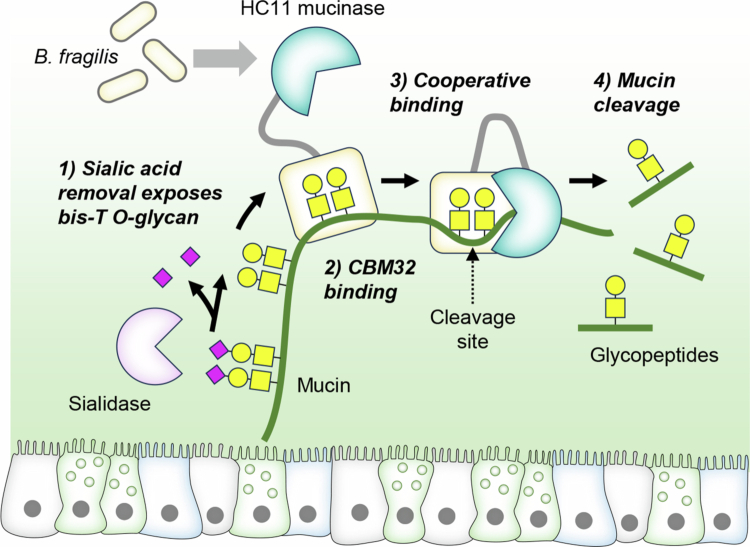
Schematic depiction of the proposed mechanism of HC11 activity in the degradation of mucins with bis-T O-glycans. First, sialidase activity deriving from *B. fragilis* or other members of the gut microbiome exposes bis-T O-glycan motifs (1) which can be bound by the CBM32 domain of the HC11 mucinase secreted from *B. fragilis* (2). Multivalent interactions of the CBM32 domain with repeat O-glycan motifs presumably orients the mucin substrate for cooperative engagement with the catalytic domain of HC11 (3) and cleavage in between the two adjacent O-glycans (4). The resulting glycopeptides may be utilized by B. fragilis for self-feeding or serve cross-feeding.

Here, our studies of a panel of nine M60-like mucinase candidates revealed two functional classes, represented by the bis-Tn mucinase *B. thetaiotaomicron* BT4244 and the bis-T–preferring mucinase *B. fragilis* HC11. These mucinases share cleavage at a bis-O-glycan dyad but differ in their preference for Tn versus T structures, guided by coordinated properties of their catalytic and CBM32 domains (Figure 7). Their specificity for bis-O-glycan substrate sites was demonstrated to be dependent on a conserved tyrosine residue (Tyr723 in BT4244 and Tyr817 in HC11) in the catalytic domains and conserved among M60 mucinases. The differences in substrate specificity for bis-Tn and bis-T O-glycans was shown to be dependent on the coordinate interactions of the catalytic and CBM32 domains. The CBM32 domains are essential for cleavage of extended mucin substrates, enabling multivalent engagement, whereas the catalytic domains alone are active with short glycopeptide substrates. Such cooperative catalytic functions of CBMs of microbial mucinases and glycosidases have been suggested previously; for example, the CBM32 module of the *B. bifidum* sulfoglycosidase BbhII enhances activity, although the mechanistic basis of this cooperativity remains unclear.^42^^,^^43^ To study the mucin-degrading properties of mucinases, we relied on our mucin reporters that are designed to encompass all the critical features of human mucins as substrates for mucinases. These mucin reporters are designed with 150–200 amino acid sequence motifs from the representative tandem repeats of mucins with their unique patterns and clustering of O-glycans. When expressed in genetically glycoengineered cells, the mucin reporters can be displayed and produced with custom-designed O-glycans with the cell-based mucin array platform.^20^ While these mucin reporters enable unprecedented dissection of the substrate specificities of mucinases, it is noteworthy that these reporters do not include the full mucins and/or their oligomeric states as found in the mucus linings. We employed site-directed mutagenesis and MD simulations to support the interpretation of the cooperative mechanism of the catalytic and CBM domains in productive substrate engagement and catalysis of extended mucin substrates. Although the MD simulations help rationalize the observed glycoform preference, they are intended as qualitative, hypothesis-generating support rather than atomic-resolution or definitive evidence of specificity, given the manual docking of substrates and the use of positional restraints.

Most mucinases identified to date, including the M60-like mucinases, only cleave mucins with eroded truncated O-glycans.^14-19^^,^^28^^,^^44^ The microbiome is equipped with a full range of glycoside hydrolases that can release monosaccharides from the elaborate O-glycans on nascent mucins to generate mucins with eroded T and Tn O-glycans.^9-11^^,^^45^ The T (core1) disaccharide O-glycans can be released by endoglycosidases,^25^^,^^46^ and the Tn O-glycans with the inner obligate αGalNAc residue attached to Ser/Thr residues by different *α*-*N*-acetylgalactosaminidases.^47-49^ The advantage of sequentially eroding O-glycans and subsequent mucinase digestion of mucins with Tn and/or T O-glycans may be that the resulting short glycopeptides can be retained and imported into the bacterial periplasmic space for further degradation and nutrient acquisition from both glycans and amino acids. Uptake of glycopeptides derived from mucinase activity may involve SusCD protein complexes on the outer membrane of *Bacteroides* and other species that have been shown to import glycans and peptides.^50-53^ Davey et al. reported that *Akkermansia muciniphila* can take up extracellular mucins and mucin degradation products into an intracellular compartment, potentially via a specialized mucin utilization loci (MUL) that encodes for pili and periplasmic protein complexes.^54^ Further studies are required to better understand the extent and underlying mechanisms by which glycopeptides generated through mucinase activity are transported. Moreover, access to GalNAc monosaccharides is important as demonstrated by studying how the blood group ABO status affects the composition of the microbiome and the availability of GalNAc.^55^^,^^56^ GalNAc monosaccharide can be sourced by gut bacteria from the terminal blood group A antigens (GalNAcα1-3[Fucα1-2]Galβ1-R)^57^ as well as SDa antigens (GalNAcβ1-4[NeuAcα2-3]Galβ1-R]^58^ on different types of glycoconjugates including mucins as well as from glycolipids and exogenous food sources.^59^ However, mucins with their obligate inner αGalNAc residues are the major and only consistent endogenous source of GalNAc monosaccharides for the gut microbiome and independent of the histo-blood group status and diet in humans. Thus, mucinases like BT4244 and HC11 that cleave mucins with partially eroded O-glycans are likely serving in the final stages of the sequential process of the microbiome grazing on mucins following removal of monosaccharides to cleave the mucin backbone into small glycopeptides that may be amenable for import and further digestion into amino acids.^9^^,^^31^ Isolated catalytic domains of M60 O-glycopeptidase family members from *B. caccae*, including BT4244 were recently reported to hydrolyze mucins carrying branched and sialylated O-glycans *in vitro*, suggesting that M60 family members may possibly target extended O-glycan substrates.^44^ However, the influence of putative MBM on the catalytic activity and substrate usage in the presence of potentially preferred eroded O-glycans should be considered. We speculate that mucinases with broader tolerance for extended O-glycans may, in certain contexts, access less-processed mucus regions, such as those closer to the inner mucus layer where newly secreted mucins retain more complex glycosylation. Enzymatic activity in this compartment could, in principle, compromise mucus barrier integrity and thereby contribute to pathology. However, such activity may also represent a component of physiological mucus turnover. The consequences of mucinase activity are thus likely to be context dependent and *in vivo* investigation is required to determine their impact on barrier function.

Here, we also demonstrated that *B. fragilis* secretes the HC11 mucinase and requires prior removal of sialic acids to degrade the native human mucins produced by human intestinal organoids. The organoid mucus likely consists of MUC2 with sialyl-T O-glycans as evidenced by labeling with X409 MBM and exposed PNA labeling following desialylation, respectively (Figure 6). The mucolytic activity of *B. fragilis* was completely lost following deletion of the HC11 gene and fully restored upon complementation, providing direct evidence for the physiological relevance of HC11 in *B. fragilis*–mediated mucin degradation at the epithelial interface. These findings support a role of HC11 activity downstream of exoglycosidases, such as sialidases, in a piecemeal degradation cascade to access the nutrient-rich mucins (Figure 7). Notably, *B. fragilis* has a described sialidase NanH^60^ and these bacteria are known to utilize sialic acid as a carbon and energy source.^61^ The *B. fragilis* sialidase was recently shown to enable utilization of human milk oligosaccharides (HMOs) and potentially mucin glycans for colonization of the infant intestinal mucosa.^62^^,^^63^ We observed evidence of sialidase activity when growing *B. fragilis* on plain medium, but not in rich BHI medium, whereas mucin-degrading activity was found under all culture condition. In agreement with these findings, transcriptomic analyzes have shown that the *B. fragilis* 638 R sialidase gene (BF638R_1728) is poorly expressed in rich medium but highly expressed in bacteria monocolonizing the mouse gut, whereas the HC11 gene (BF638R_2945) is expressed by bacteria grown in rich medium as well as in the monocolonized mouse gut.^64^ Genes encoding the sialidase and the HC11 mucinase are core genes of *B. fragilis* as would be expected of an organism that relies substantially on glycosylated mucins as a nutrient source.^65^ However, mucin degradation is a cooperative process involving multiple members of the gut microbiome.^8-11^ Several bacteria species produce sialidases and release sialic acids from mucins for their own use or for cross-feeding, thereby exposing underlying bis-T O-glycan structures.^66^ Therefore, *B. fragilis* may not require its own sialidase to facilitate HC11 mucinase activity as it could instead utilize mucin substrates generated by sialidases from other bacteria. Likewise, glycopeptides generated through HC11 activity may also be utilized by other members of the gut microbiome. It remains to be determined whether HC11 activity provides *B. fragilis* with a competitive advantage in accessing mucin-derived nutrients for self-feeding or whether it primarily contributes to collective mucin degradation at the community level.

Our discovery strategy for mucinases employs a modular CBM-guided walk, leveraging the multimodular nature with CBMs for most of these microbial enzymes.^28^^,^^30^ CBMs likely assist targeting in mucus, provide multivalent avid interactions, and orient substrates for cleavage on repeat O-glycan motifs.^12^ Here, we showed the critical importance of CBMs for the function of M60-like mucinases (Figure 4) and highlighted evidence for cooperative substrate interactions between the catalytic domain and the CBMs. The catalytic sites of these mucinases clearly recognize adjacent bis O-glycans, but mutational analysis revealed that the catalytic domains alone do not bind mucins or O-glycans effectively, whereas binding was observed with the CBM32 modules and interestingly markedly enhanced with the full mucinases (Figure 5). Notably, CBMs were required for cleavage of large mucin fragments in solution and on cells, but not for short glycopeptides, which is consistent with a multivalency-dependent mechanism. Therefore, CBM32 modules may serve in orienting the mucin substrate in a geometry favorable for cleavage by the catalytic domain in bis-O-glycan motifs. Such cooperative mechanism is reminiscent of that of eukaryotic GALNTs that initiate mucin-type O-glycosylation.^67^ GALNTs have C-terminal CBM13 modules that mediate binding to partially GalNAc-glycosylated glycopeptide substrates to facilitate glycosylation at distant Ser/Thr glycosite positions.^68^^,^^69^ The GALNT CBM13 domains are indispensable for glycosylation of select O-glycosites in proteins, but not for the general catalytic functions and glycosylation of short glycopeptides.^70^^,^^71^ A similar scenario was reported for an exo-acting *β*-fructosidase.^72^ Notably, the essential role of CBMs demonstrated here differs markedly from other glycoside hydrolase systems reported previously,^32^ e.g., as a recently described sulfoglycosidase, where mutations in the CBM resulted only in minor reduction of enzymatic activity.^42^ In contrast, CBM32 in HC11 and BT4244 is essential for processing physiologically relevant extended substrates, indicating a specialized adaptation for mucus-rich environments.

CBM32 and CBM51 are widely found on M60-like mucinases.^30^ Studies of the *Clostridium perfringens* ZmpA, ZmpB, and ZmpC multidomain mucinases appended with both CBM32 and CBM51 previously demonstrated that these recognize GalNAc and GlcNAc residues, respectively, and it was suggested that they serve in substrate targeting rather than catalysis.^12^ ZmpC and ZmpB were crystallized with sialylated T antigens (mSTb), and although both CBMs and catalytic domains bind O-glycans, no experimental evidence supports cooperative substrate recognition between the domains.^12^ M60-like mucinases are also found without CBMs appended like the AM0627 mucinase, which we and others previously characterized as a bis-T/Tn mucinase that in solution assays efficiently cleaves extended mucin reporters, different glycoproteins, and short glycopeptides.^17^^,^^24^ Here, surprisingly we found that the AM0627 mucinase exhibited negligible cleavage of mucin reporters when displayed on the surface of cells but not with the same reporters in solution (Supplementary Figure 3). This presents a third pattern of activity of mucinases with extended mucin substrates and although this finding clearly requires further studies, it is conceivable that the lack of CBMs on the AM0627 mucinase plays a role.

The truncated Tn and T O-glycans are prominent cancer-associated glycans, and mucins and other mucin-like proteins with these O-glycans are found on the surface of cancer cells.^73^^,^^74^ Mucinases may find use in cancer therapy as suggested by the Bertozzi team, who used nanobody delivery of an attenuated variant of the enteropathogenic StcE mucinase to degrade the cell surface mucin-rich glycocalyx on cancer cells.^75^ Similarly, Paszek and coworkers showed that surface-displayed StcE on natural killer cells enhanced cell-mediated cytotoxicity against mucin-expressing cancer cells.^76^ StcE is capable of cleaving mucins and mucin-like proteins with nascent complex glycans and hence may have adverse effects without efficient targeting to cancer cells.^20^ Mucinases like BT4244 and HC11 that only cleave mucins with the truncated Tn and T O-glycans that are only found on cancer cells may offer safer alternatives. However, more recently Bertozzi and colleagues demonstrated similar release of cell surface mucins by use of the human cathepsin K protease.^77^

In conclusion, our study identifies two functional classes of M60-like mucinases with distinct bis-Tn and bis-T specificities and provides the first direct evidence that CBM32 is indispensable for efficient cleavage of extended mucin substrates (Figure 7). These findings redefine the catalytic architecture of M60 mucinases and uncover previously unrecognized mechanistic cooperativity between CBMs and catalytic domains. They also situate BT4244 and HC11 within the late stages of mucin turnover by gut symbionts, underscoring their role in accessing mucin-derived nutrients.

## Materials and methods

### Production of M60-like mucinases

A panel of M60-like mucinases identified in this study was produced as described previously.^28^ The M60-like mucinase candidates were denoted HC10-20 for consistent naming with previously reported mucinases identified with our molecular walk strategy.^28^ Briefly, codon optimized sequences for full coding variants of HC10-HC20 (HC12 represents BT4244) without recognizable signal peptides were obtained from Twist Bioscience. Sequences were cloned into a pET28-based vector and transformed into T7 Express (NEB) bacterial strains grown at 37 °C for 2 h with induction by 1 mM IPTG for 30 min. Cultures were continued to grow at 16 °C overnight and bacteria were harvested by centrifugation and lysed in buffer A (50 mM Tris-HCl, 300 M NaCl, 10 mM imidazole, pH 8) supplemented with 0.5 mg/ml lysozyme. The expression constructs used for BT4244, HC7, and StcE were reported previously.^17^^,^^20^^,^^28^ Proteins were purified using Ni Sepharose 6 Fast Flow resin (GE Healthcare) and eluted with buffer B (Buffer A + 250 mM imidazole). Fractions containing enzymes were pooled, dialyzed in 1× PBS, and frozen. Mutants of HC11 (W255/R285A) and BT4244 (W171/R199A) were generated by GenScript via site-directed mutagenesis of the vector pMALC2x-12Hist-TEV-HC11 as described previously^28^, and all the other site-directed mutagenesis were performed with the PrimeSTAR Mutagenesis Basal Kit (Takara Bio). The mutants were purified using the same protocol as for AM0627.^17^ Proteins were concentrated using Amicon Ultra-15 mL and quantification was carried out by absorbance at 280 nm using their theoretical extinction coefficients.

### Glycoengineered cells

HEK293 wild type (WT) and a panel isogenic cell lines glycoengineered by stable knockout/knockin (KO/KI) of glycosyltransferase genes, as previously reported were used.^20^^,^^37^ HEK293^WT^ cells express a mixture of sialylated core1/2 structures, HEK293^KO *GCNT1*^ produce dST, HEK293^KO *GCNT1*, *ST6GANAC2/3/4*^ produce mSTa, HEK293^KO *GCNT1, ST3GAL1/2, ST6GANAC2/3/4*^ produce T (core1), HEK293^KO *C1GALT1*^ produce Tn, HEK293^KO *COSMC*, KI B3GnT6^ produce core3, and HEK293^KO *COSMC*, KI ST6GalNAc1^ produce STn. The cells were cultured in DMEM (Sigma-Aldrich) supplemented with 10% heat-inactivated fetal bovine serum (Sigma-Aldrich) and 2 mM GlutaMAX (Gibco). The cells were maintained in a humidified incubator at 37 °C and 5% CO_2_. Suspension-adopted HEK293 cells were cultured in Freestyle F17 medium supplemented with 0.1% Kolliphor *P* 188 (Sigma-Aldrich) and 4 mM GlutaMAX in a humidified incubator at 37 °C and 5% CO_2_ on an orbital shaker under constant agitation (120 rpm).

### Production of mucin reporters

Transmembrane and secreted mucin reporters were produced as previously described.^20^ Briefly, mucin reporters for cell surface expression were generated by fusion of a MUC1 signal peptide (amino acids 1-62, Uniprot P15921) with a FLAG-tag (DYKDDDDK) and an enhanced green fluorescent protein (EGFP) sequence (*N*-terminus) followed by a human mucin O-glycodomain sequence of interest (around 150–200 amino acids), and the membrane anchoring domain of human MUC1 (amino acids 1042–1138) (C-terminus). For secreted mucin reporters, the C-terminal MUC1 membrane anchoring domain was replaced by a 6xHis tag sequence and were produced in glycoengineered isogenic HEK293 as described previously.^20^ Briefly, cells stably expressing mucin reporters were seeded at a density of 0.25 × 10^6^ cells/ml and cultured for 5 d on an orbital shaker, and the reporters were purified from the culture medium by Ni-NTA affinity (Qiagen) chromatography (pre-equilibration with 25 mM sodium phosphate, 0.5 M NaCl, 10 mM imidazole pH 7.4, and elution with 200 mM imidazole). Buffer exchange to MilliQ was performed with Zeba™ spin desalting columns (Thermo Fisher Scientific) and yields were quantified using a Pierce™ BCA protein assay kit (Thermo Fisher Scientific).

### Cell-based mucin array analysis of mucinase activity and binding properties

Cell-based mucinase cleavage assays were performed with HEK293 cells transiently expressing transmembrane mucin reporters as previously described.^28^ Briefly, 0.3 × 10^6^ adherent cells were seeded in 6-well plates and after 24 h, cells were transfected with 1.5 µg plasmid encoding different mucin reporters encoding various mucin reporters using eLipofectamine™ 3000 reagent (Thermo Fisher Scientific), following the manufacturer's instructions. After 24 h, cells were harvested and incubated with purified mucinases (0–1 µg/ml) in PBA (1× PBS, 1% BSA (w/v)) for 1 h at 37 °C, followed by washing with PBA and staining with 0.2 µg/ml APC-conjugated rat anti-FLAG antibody (clone L5, BioLegend) for 30 min at 4 °C. After another wash with PBA, cells were analyzed using a Sony SA3800 spectral analyzer. For cells transiently expressing reporters, binding of the anti-FLAG antibody was measured both GFP positive (reporter transfected) and negative (non-transfected) populations and Mean fluorescence intensity (MFI) was quantified by using FlowJo software (LLC). Cell-based mucin binding assays were performed with HEK293 cells transiently expressing mucin reporters. Cells were incubation with isolated, catalytically inactive mucinases CBMs for 1 h at 4 °C, followed by staining with Alexa Fluor 647-conjugated anti-6xHis antibody (R&D). MFI of anti-6xHis antibody binding was determined in GFP positive(transfected) cells expressing membrane-bound mucin TR reporters and compared to GFP negative (non-transfected) control cells, using FlowJo (FlowJo LLC).

### In solution mucinase assays with secreted isolated mucin reporters

Purified mucinases were incubated with 500 ng purified mucin reporters bearing different O-glycans at varying enzyme to substrate ratios (1:25 to 250) in a 20  µl reaction volume of 50 mM ammonium bicarbonate buffer. Reactions were carried out for 1 h at 37 °C and then stopped by heat-inactivation at 95 °C for 5 min. Samples were separated on 4–12% Bis-Tris gels (NuPAGE, Novex) at 100 V for 1 h and gels were stained using Krypton Fluorescent Protein Stain (Thermo Fisher Scientific) according to the manufacturer's instructions and imaged with an ImageQuant LAS 4000 system (GE Healthcare).

### Solid-phase GalNAc glycopeptide synthesis (SPPS) and enzyme cleavage assay

The Tn 16-mer MUC1 glycopeptide (APGS*T*APPAHGVT*S*AP, * indicates GalNAc) and 10-mer PSGL1 glycopeptide (TEAQT*T*PPPA, * indicates GalNAc) were synthesized by stepwise microwave-assisted solid-phase synthesis on a Liberty Blue synthesizer using the Fmoc strategy on Rink Amide MBHA resin (0.1 mmol). Fmoc-Thr[GalNAc(Ac)3-*α*-D]-OH (2.0 equiv) and Fmoc-Thr(GalN3(Ac)3-*α*-D]-OH (2.0 equiv) were synthesized as described previously^17^ and manually coupled using HBTU [(2(1H-benzotriazol-1-yl)-1,1,3,3-tetramethyluronium hexafluorophosphate], while all other Fmoc amino acids (5.0 equiv.) were automatically coupled using oxyma pure/DIC (*N*,N’-diisopropylcarbodiimide). The O-acetyl groups of GalNAc moieties were removed in a mixture of NH2NH2/MeOH (7:3). Glycopeptides were then released from the resin, and all acid-sensitive side-chain protecting groups were simultaneously removed using TFA 95%, TIS (triisopropylsilane) 2.5% and H2O 2.5%, followed by precipitation with cold diethyl ether. The crude products were purified by HPLC on a Phenomenex Luna C18(2) column (10 μm, 250 mm × 21.2 mm) and a dual absorbance detector, with a flow rate of 10 mL/min. Glycopeptides (1 μg) in 100 mM in 25 mM Tris (pH 7.5) were incubated with serial dilutions of enzymes at 37 °C for 1 h in 50 mM ammonium bicarbonate (pH 8.0). Reactions were terminated by 0.1% TFA and product development monitored and semi-quantified by MALDI-TOF MS. The T-glycopeptides were enzymatically synthesized from the Tn-glycopeptides using the *Dm*C1GalT1 core1 synthase^78^ as described before.^17^ Cleavage assays were performed using synthesized glycopeptides (1 μg) initially dissolved in 25 mM Tris buffer (pH 7.5). The peptides were incubated with serial dilutions of enzymes (12.5 ng–1 μg) at 37 °C for 1 h in 50 mM ammonium bicarbonate (pH 8.0). Reactions were terminated by the addition of 0.1% trifluoroacetic acid (TFA), and product formation was monitored and semi-quantified using MALDI-TOF mass spectrometry.

### Mass photometry analysis

Molecular mass distributions were characterized using mass photometry (TwoMP, Refeyn). Immediately prior analysis, No. 1.5 H glass coverslips (Paul Marienfeld GmbH) were sequentially rinsed five times with Milli-Q water and HPLC-grade isopropanol, then dried with filtered air. Silicon gaskets were then applied to the cleaned glass to create sample wells. The instrument was calibrated using a Native protein marker (Invitrogen) covering a range of 66 to 1048 kDa. Initial quality checks were performed by diluting protein stocks to 100 nM in PBS, with final measurements taken at 5 nM. For binding assays, 5 nM of either mucin or O-Glycocarrier reporters were incubated with 50 nM MBP-X409 in a 20 µL volume for 15 min at room temperature. Each measurement was conducted in a fresh gasket well. Data were captured for 90 s using AcquireMP (Refeyn Ltd) once autofocus had stabilized. Analysis was performed in DiscoverMP (Refeyn Ltd), with results visualized as kernel density estimates (5 kDa bandwidth). Uncertainties were calculated based on Gaussian curve fitting errors within the DiscoverMP suite.

### Molecular dynamics (MD) simulation

MD simulations were performed using the AMBER 20 package,^79^ employing the ff14SB^80^ and GLYCAM06j-2^81^ force fields. Protein structures predicted by AlphaFold served as the basis for the models, with glycopeptides manually fitted into the enzyme active site and the CBM. Each system was solvated in a truncated octahedral box of TIP3P water molecules, maintaining a 10 Å buffer around the solute. Explicit counterions were added to neutralize the systems. A two-stage energy minimization protocol was employed: first, restraining solute atoms while minimizing solvent molecules, followed by a full unrestrained minimization of all atoms in the simulation box. Subsequently, the systems were gradually heated from 0 to 300 K under constant pressure (1 atm) and periodic boundary conditions. Harmonic restraints of 30 kcal·mol⁻¹·Å⁻² were applied to the solute, and temperature regulation was achieved using the Andersen thermostat. A 1 fs time step was used during heating to allow for smooth thermal equilibration. Long-range electrostatics were treated using the particle mesh Ewald (PME) method,^5^ and an 8 Å cutoff was applied to Lennard‒Jones interactions. Equilibration was performed for 2 ns under constant volume and temperature (NVT ensemble) at 300 K, using a 2-fs time step. Production runs of 300 ns were then carried out under the same conditions, with positional restraints applied to maintain the glycan moiety within the active site and the CBM (see Supplementary Figures 8 and 9). Accordingly, the restrained simulations were designed to assess the persistence of plausible contacts in a predefined binding orientation.

### *B. fragilis* culture

*B. fragilis* strains NTCT9343 and 86-5433-2-2 strains and deletion mutants used in this study are listed in Supplementary Table 1. The bacteria were cultured in BHI broth (BD) supplemented with 5 g/L yeast extract (BD), 0.5 g/L L-cysteine (Sigma-Aldrich), 10 ml/L Hemin solution (Sigma-Aldrich) and 0.2 mL/L Vitamin K_1_ (Sigma-Aldrich). Bacteria were grown over night, reinoculated at an O.D.600 of 0.05 and grown to an O.D.600 of 0.4. For all downstream experiments, bacteria strains (NTCT9343 WT, ΔHC11 and ΔHC11+HC11; 86-5433-2-2 WT, ΔHC11 and ΔHC11+HC11) were washed twice with PBS, diluted 10× in supplemented BHI broth or M9 medium (1 g/L NH_4_Cl, 6 g/L Na_2_HPO_4_, 3 g/L KH_2_PO_4_, 0.5 g/L NaCl, 0.1 mM CaCl_2_·2H_2_O, 1 mM MgSO_4_·7H_2_O, 0.05% L-Cysteine, 5 mg/mL Hemin, 2.5 mg/mL VitK_1_, 2 mg/mL FeSO_4_·7H_2_O, 5 ng/mL VitB_12_ (Sigma-Aldrich)) with 0.05% glucose, and 5 µg of the different MUC1 and MUC2 TR reporters. All cultures were performed in anaerobic conditions using a BACTRON Anaerobic Chamber (VWR) or Concept 500 (Baker Ruskinn) at 37 °C.

### Construction of HC11 gene deletions in *B. fragilis* and complementation

All the plasmids and primers used in this study are listed in Supplementary Table 1. All the plasmids were verified by whole plasmid sequencing. The PCR products were amplified for cloning using Phusion polymerase (NEB), and NEBuilder (NEB) was used to join all DNA pieces. Deletions in *B. fragilis* NCTC9343 and *B. fragilis* 86-5443-2-2 were constructed by PCR amplifying regions upstream and downstream of the target gene. The DNA regions of 9343 were cloned into the BamHI site of pLGB13^82^ and the DNA regions of 86-5443-2-2 were cloned into pMLS35, a plasmid in which the *ermG* of pLGB13 is replaced with *cfxA*. Recombinant plasmids were conjugally transferred from *E. coli* S17 *λ* to *B. fragilis*. Cointegrates were selected on BHIS plates containing gentamycin with either erythromycin (10 μg/ml) or cefoxitin (10 μg/ml). Cointegrants were passaged in basal media for several hours and plated onto BHIS plates containing anhydrotetracycline (75 ng/ml) to select for double cross out recombinants. Mutants were identified by PCR. For complementation of *B. fragilis* NCTC9343 ΔBF9343_2853, this gene with its native promoter was cloned into the BamHI site of pNBU2-*bla-ermG*.^83^ This plasmid was transferred into *B. fragilis* NCTC 9343 ΔBF9343_2853 where it integrated into the *attB* site. For complementation of *B. fragilis* O86-5443-2-2 ΔAC141_31010, plasmid pKF55 was first created where *ermG* of pNBU2-*bla-ermG* was swapping with *cfxA* from *P. vulgatus* CL11T00C01.^82^ AC141_31010 with its upstream promoter was cloned into the BamHI site of pKF55. This plasmid was transferred into O86-5443-2-2 ΔAC141_31010 where it integrated into the *attB* site. Integrants in 9343 or 86-5443-2-2 were selected on BHIS plates containing gentamycin with either erythromycin or cefoxitin.

### Colon organoid 3D expansion and colonic mucus generation in ALI 2D organoid culture

Ethical approval was obtained from the ethics committees of the University Medical Center Utrecht. All experiments and analyzes were performed in compliance with relevant ethical regulations. Organoids were expanded in 3D domes of Cultrex Pathclear Reduced Growth Factor Basement Membrane Extract (BME) (3533-001, Amsbio), with expansion medium containing Advanced DMEM/F12 (Gibco), 1× B27, 1× glutamax, 10 mmol/l HEPES, 100 U/ml penicillin-streptomycin (all Thermo-Fisher), 1.25 mM *N*-acetylcysteine, 10 μM nicotinamide, 10 μM p38 inhibitor SB202190 (all Sigma-Aldrich) and the following growth factors: 0.5 nM Wnt surrogate-Fc fusion protein, 2% noggin conditioned medium (both U-Protein Express), 20% Rspo1 conditioned medium (in-house), 50 ng/ml EGF (Peprotech), 0.5 μM A83-01, and 1 μM PGE2 (both Tocris).^84^ Colonic mucus was generated and harvested from 2D cultures of colon organoids as follows: 100.000 single cells were seeded per 24 well transwell (Greiner, 662640) precoated with 5% BME for 30 min at 37 °C. Cells were grown to confluency, typically 2–3 d, in expansion medium supplemented with ROCK inhibitor Y-27632 (10 μM; Abmole, M1817) during the first 2 d. The medium was removed from the upper compartment, and cells were grown in air liquid interface (ALI) for 4 d. Afterwards, medium from the bottom compartment was exchanged for differentiation medium (expansion medium without R-spondin, WNT-surrogate, *N*-acetylcysteine and supplemented with 10 μM DAPT (Sigma-Aldrich, D5942)) during 4 extra days. Mucus was collected in 100 µl of PBS per transwell.

### *B. fragilis* mucinase assay

*B. fragilis* strain NTCT9343 WT and *Parabacteroides distasonis* strain DSM20701 were inoculated in supplemented BHI broth (Merck) at and O.D.600 of 0.05 and grown overnight in anaerobic conditions at 37 °C. Per condition, 1 mL of culture was spun down at 3000 × g for 5 min, and the supernatant was filter-sterilized using a 0.22 µm filter. Forty-five microliters of the supernatants were incubated with 5 µg of the different MUC2 TR reporters, with or without 150 mU/mL *C. perfringens* sialidase (Roche). Incubation was performed during 8 or 24 h at 37 °C. The samples were run in 4–15% Mini-PROTEAN® TGX™ Precast Protein Gels (Biorad) and transferred into PVDF membranes. Blots were stained overnight at 4 °C with 1 µg/mL mouse anti-FLAG M2 antibody (Sigma-Aldrich) followed by incubation with 0.1 µg/mL rabbit anti-mouse-HRP (Dako) for 2 h at room temperature. The membranes were imaged using the Amersham ImageQuant 800 western blot imaging system. Alternatively, *B. fragilis* strains (NTCT9343 WT, ΔHC11 and ΔHC11+HC11; 86-5433-2-2 WT, ΔHC11 and ΔHC11+HC11) were grown in supplemented BHI medium to an O.D.600 of 0.4 and diluted 10× in fresh supplemented BHI medium together with 5 μg of MUC2 or MUC1 TR reporters with WT, core1 or Tn O-glycans or mucus (10 μl) for 24 h. For the mucus experiment, the bacteria were reinoculated in minimal M9 medium with 0.05% glucose. Optionally, 150 mU/ml *C. perfringens* sialidase (Roche) was added to the culture. The cultures were centrifuged for 5 min at 500 × g, and the supernatant was collected to analyze the mucin cleavage. For mucin TR reporters, SDS-PAGE/western blot analysis was performed and nitrocellulose membranes were stained for 1 h with 1 µg/ml mouse anti-FLAG M2 antibody (Sigma Aldrich) followed by incubation with 1 µg/ml IRDye800-conjugated anti-mouse IgG (LI-COR). For organoid-derived mucin, samples were separated by 5% Tris-Acetate gels and blotted onto PVDF membranes followed by staining for 2 h at RT with 10 µg/ml GFP-tagged X409 MBM, a previously described mucin-binding probe derived from the mucinase StcE from *E. coli* O157:H7.^20^ Additional staining was performed for 1 h at RT with 1 µg/ml biotinylated PNA lectin (Vector Laboratories) that recognizes core1 O-glycans followed by staining with 1 µg/ml AF647 conjugated streptavidin (Invitrogen). Membranes were imaged using a fluorescence imager (G:Box Chemi XX6, Syngene and Odyssey, LI-COR).

## Supplementary Material

SI Table 1.xlsx SI Table 1.xlsx

SI document final.docx SI document final.docx

## Data Availability

The authors confirm that the data supporting the findings of this study are available within the article [and/or] its supplementary materials.
