# Supplementary material for: Discovery of a secreted Bacteroides fragilis mucinase that cleaves mucins with bis-T O-glycans through a carbohydrate binding module-dependent mechanism
Source: Gut Microbes. 2026 Mar 19;18(1):2644983. doi: 10.1080/19490976.2026.2644983 (PMC13003899; doi:10.1080/19490976.2026.2644983)
Supplement: SI document final.docx [file KGMI_A_2644983_SM8291.docx]

Supplementary Materials

**Discovery of a secreted *Bacteroides fragilis* mucinase that cleaves mucins with bis-T O-glycans**

**through a carbohydrate binding module-dependent mechanism**

Yoshiki Narimatsu†^,^*, Cayetano Pleguezuelos-Manzano†, Daniël L.A.H. Hornikx†, Felix Goerdeler, Thapakorn Jaroentomeechai, Katia Flores, Sanae Narimatsu, Charelle Boot, Lars Hansen, Fabien Durbesson, Renaud Vincentelli, Laurie E. Comstock, Hans Clevers, Victor Taleb, Francisco Corzana, Bernard Henrissat, Henrik Clausen, Ramon Hurtado-Guerrero, Christian Büll*

* Corresponding author: Y.N. (yoshiki@sund.ku.dk) and C.B. (christian.bull@ru.nl)

† These authors contributed equally.

**Supplementary Figures and Tables in this study**

**Supplementary Figure 1.** Putative M60 recombinant mucinases expressed in E.coli.

**Supplementary Figure 2.** Screen of putative M60 mucinases with MUC2 mucin TR reporters.

**Supplementary Figure 3.** AM0627 cleaves secreted but not cell surface mucin(-like) domains.

**Supplementary Figure 4.** Full sequence of the mucin TR included in the reporters used in this study.

**Supplementary Figure 5.** Cleavage of synthetic O-glycan cluster reporters by BT4244, HC11, and AM0627 mucinases.

**Supplementary Figure 6.** Carbohydrate-binding module drives mucinase activity.

**Supplementary Figure 7.** Complex formation between mucin(-like) reporters and catalytically inactive BT4244 and HC11 mucinases.

**Supplementary Figure 8.** Representative frames from 300 ns restrained MD simulations of the complex with HC11/Tn-MUC1.

**Supplementary Figure 9.** MD simulations for the complexes studied in this work.

**Supplementary Figure 10.** Mucinase activity in anaerobic *B. fragilis* and *P. distasonis* cultures and conditioned medium.

**Supplementary Figure 11.** HC11 is a core1 mucinase in *B. fragilis* 86-5443-2-2.

**
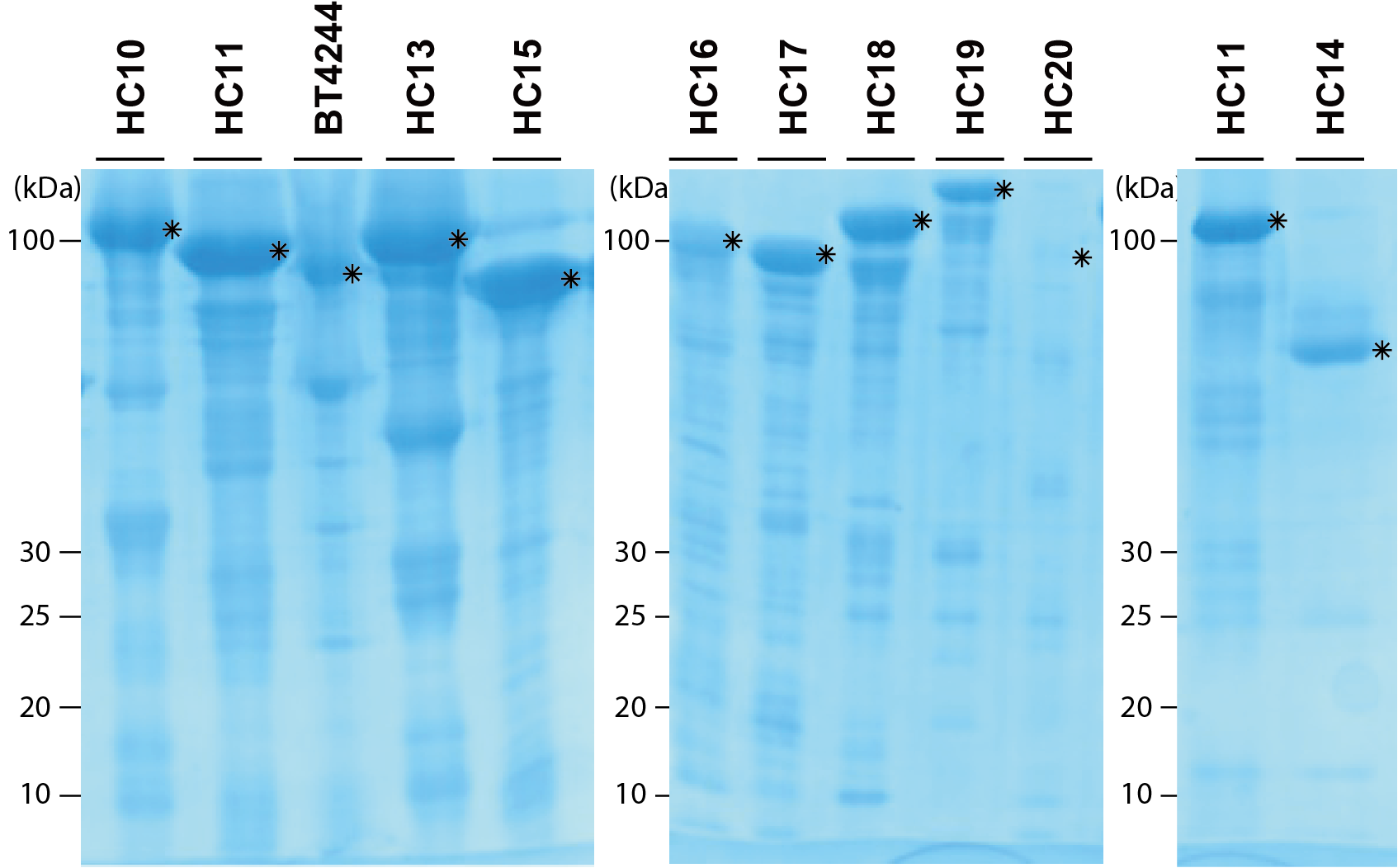
**

**Supplementary Fig. 1. Putative M60 recombinant mucinases expressed in *E. coli.*** NuPAGE analysis of the recombinant M60 mucinases following Ni affinity purification. Asterisks indicate migration of the predicted enzymes. Note that HC20 did not express well and purification failed.

**
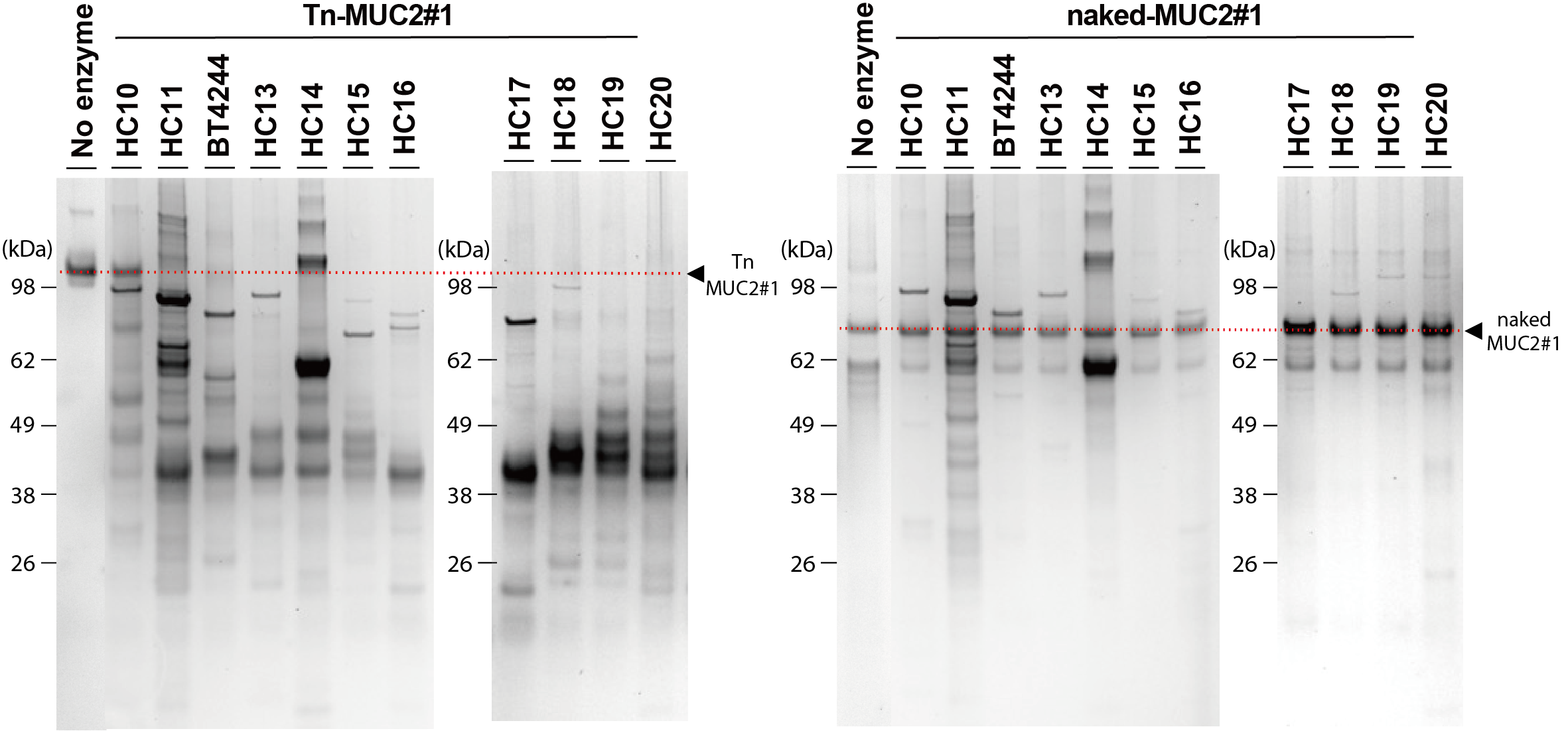
**

**Supplementary Fig. 2. Mucinase activity screen of putative M60 mucinases using isolated MUC2 mucin TR reporters.** NuPAGE analysis of the cleavage of purified MUC2#1 TR reporters (0.5 μg), produced in HEK293^KO^ *^C1GALT1^* cells (Tn-MUC2; bearing Tn O-glycans) and CHO^KO^ *^Galnt1/2/4/7/10/11/13^* (naked-MUC2; lacking O-glycans). This experiment was performed once. Migration of SeeBlue™ Plus2 pre-stained mw standards is indicated. Migration of the intact Tn-MUC2 and intact naked-MUC2 is indicated by arrows and red dashed lines.

**
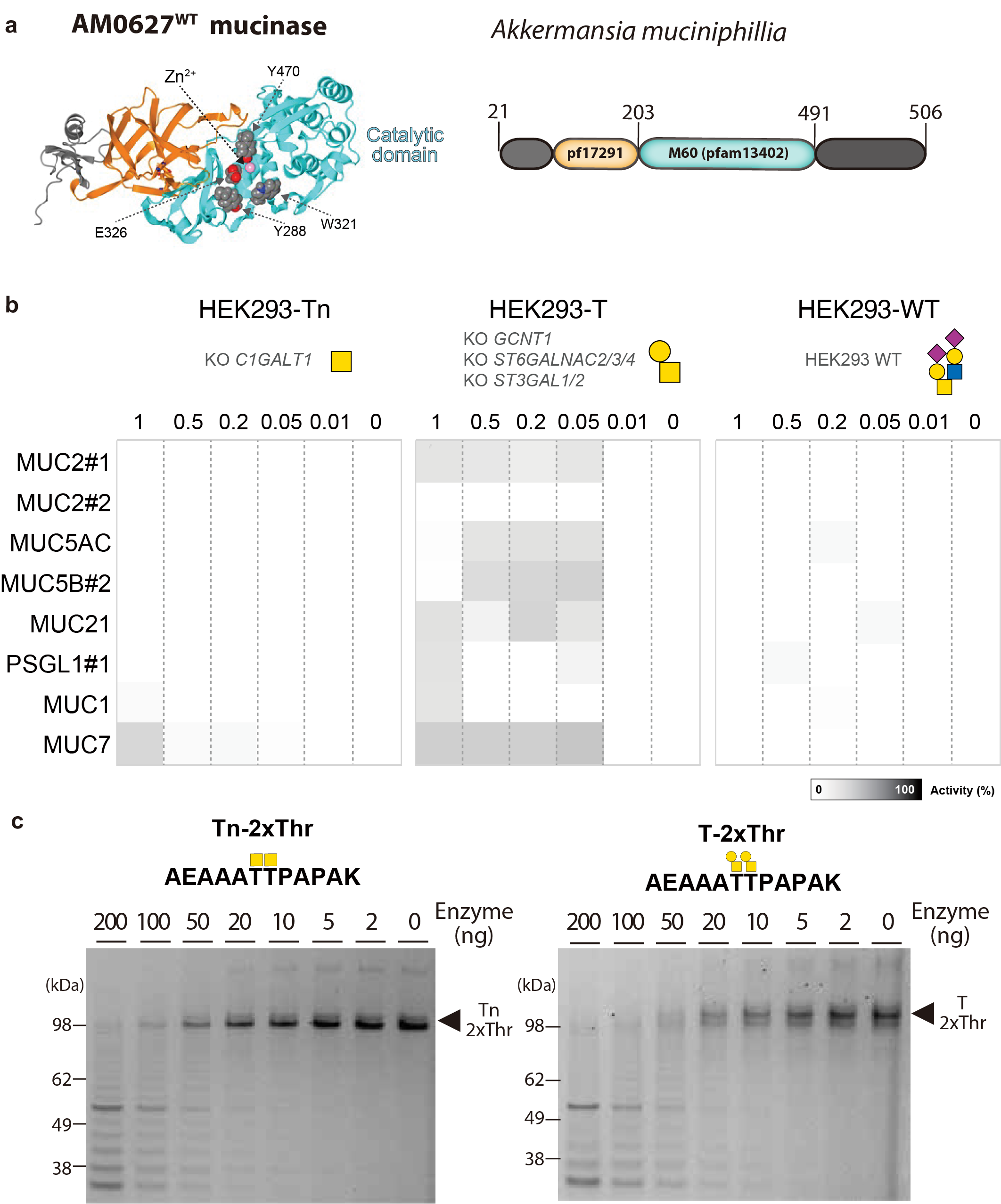
Supplementary Fig. 3. The AM0627 mucinase cleaves secreted but not cell surface displayed mucins. a)** Predicted structure (AlfaFold2 modeling) of AM0627 mucinase containing the pf17291 domain (orange), and M60 protease family domain (cyan) and schematic presentation of the modular organization of AM0627 with an M60 protease family domain (pf17291, pfam13402) and an unannotated C-terminal sequence without known function. **b)** Flow cytometry analysis of AM0627 cleavage of FLAG-tagged transmembrane mucin reporters expressed in HEK293-Tn, HEK293-T(core1), and HEK293-WT cells. The heat map shows representative activity calculated based on the MFI of anti-FLAG tag binding normalized to the respective untreated HEK293 cell lines expressing each mucin reporter construct as mean fluorescence intensity (MFI). **c**) Digestion of soluble Tn- and T/core1-2xThr reporters produced in HEK293-Tn and HEK293-T cells, respectively, by increasing concentrations of AM0627. Purified mucin reporters (500 ng) were incubated with enzymes (0–200 ng dose titration) for 1 h at 37°C and separated by Bis-Tris 4–12% gels followed by staining with Krypton fluorescent protein stain. Gels are representative of two independent experiments.

**
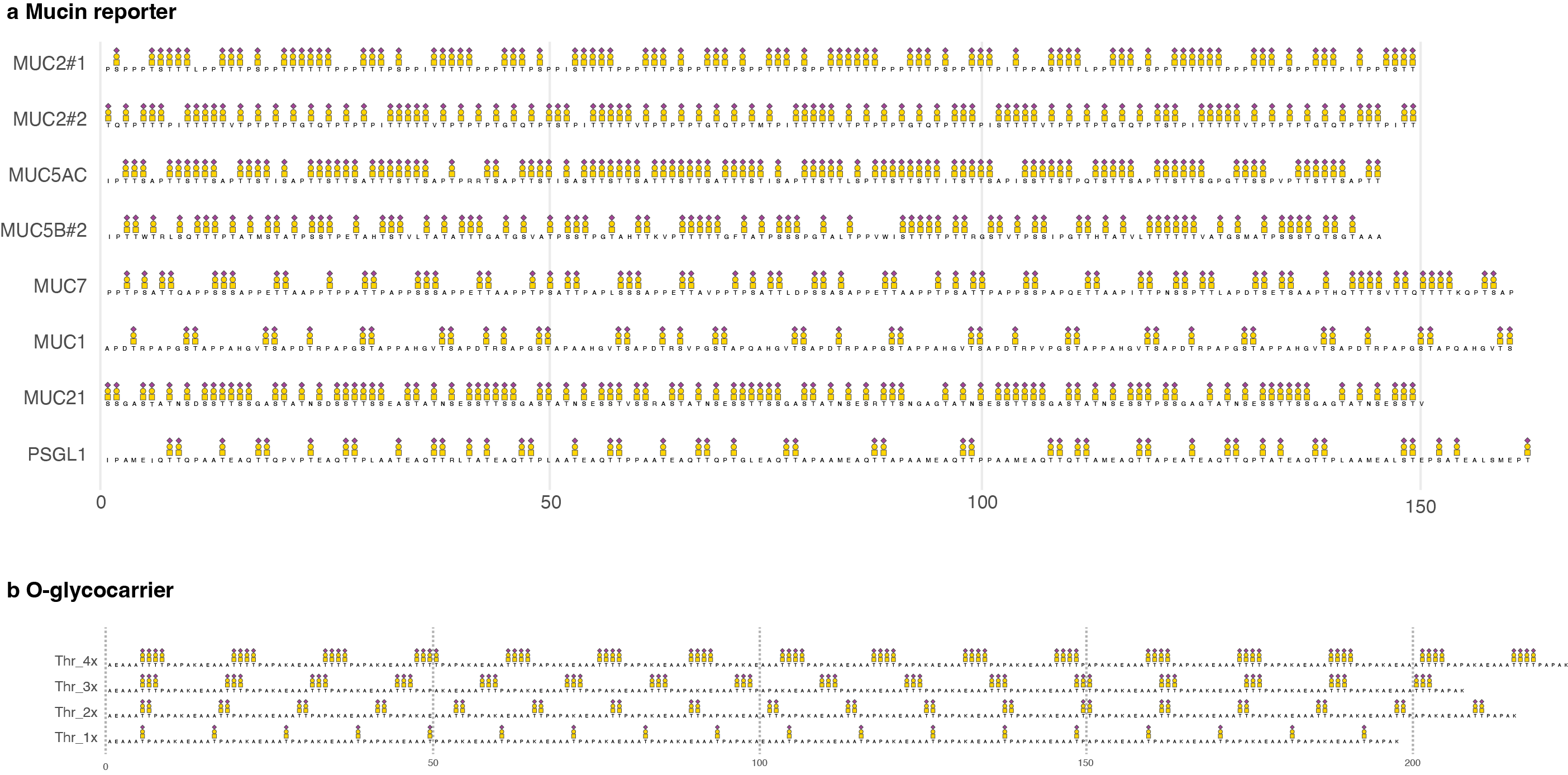
Supplementary Figure 4.** Full sequence the mucin TR included in the reporters used in the study derived from human mucins, mucin-like domains in O-glycoproteins (a) and a and the control O-glycocarrier (b). All potential Ser/Thr O-glycosites are illustrated with a trisaccharide mSTa O-glycan.

**
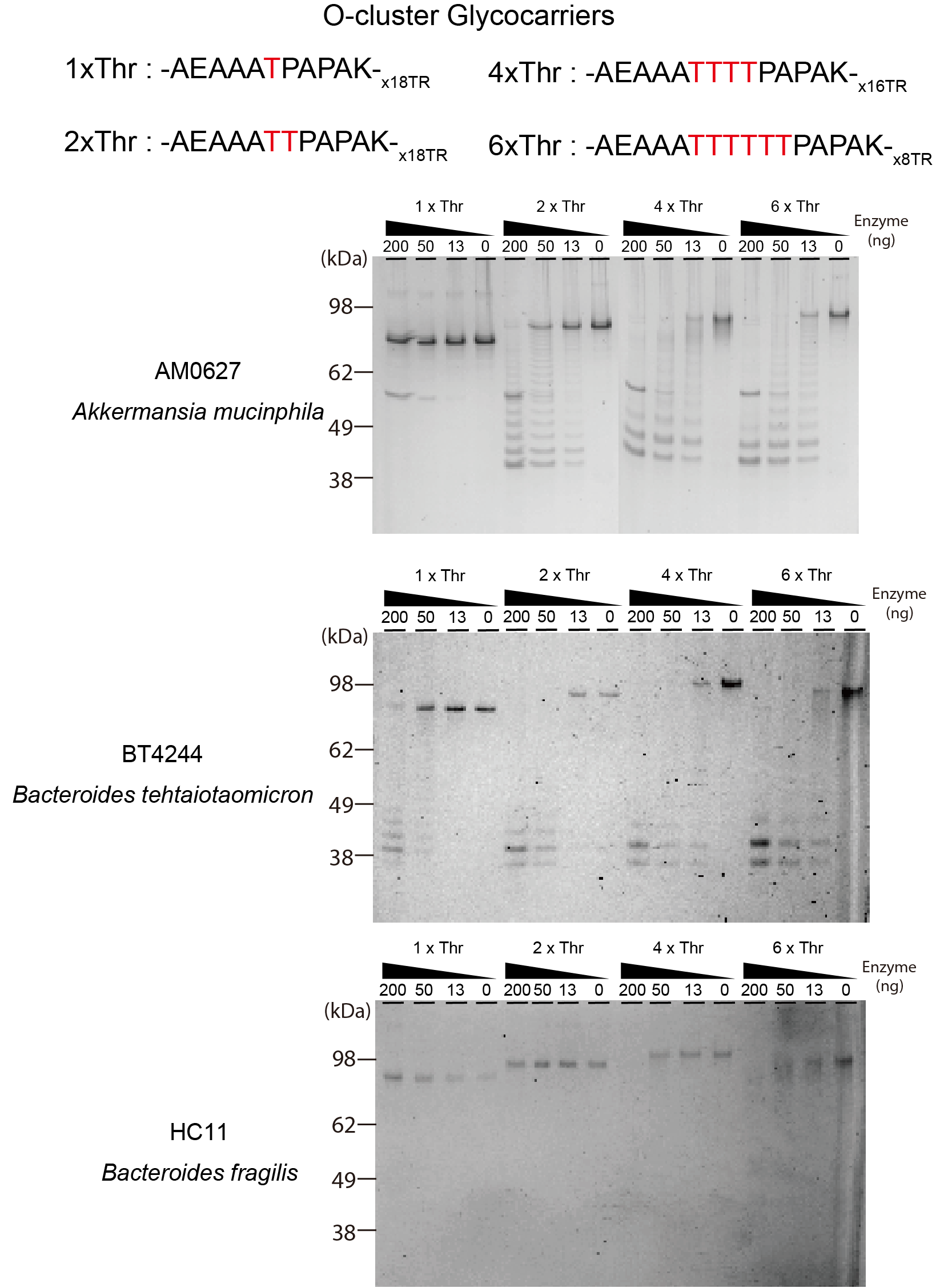
**

**Supplementary Fig. 5. Screening O-glycan clustering effect on BT4244, HC11, and AM0627 mucinase activities.** O-Glycocarrier reporters containing 8–18 tandem repeats of short peptide motifs with 1×, 2×, 4×, or 6× clustered O-glycosylation sites on threonine residues (shown in red), were expressed in HEK293-Tn cells to enable decoration with Tn O-glycans. NuPAGE analysis of cleavage of Glycocarrier reporters by AM0627, BT4244 and HC11 mucinases at different enzyme doses (0 to 200 ng). The experiment was performed once.


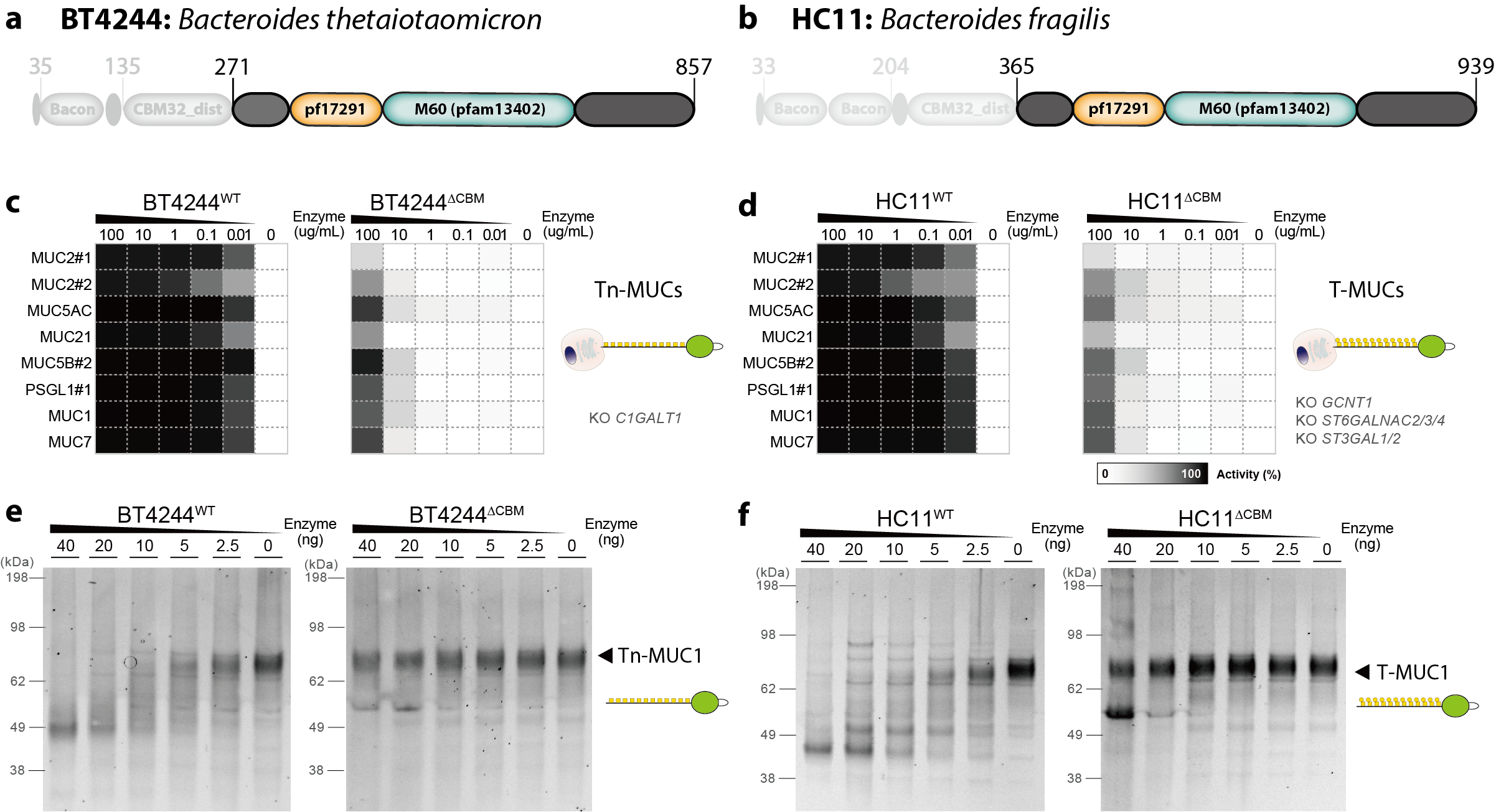


**Supplementary Fig. 6. BT4244 and HC11 require their CBM32 module to digest mucins. a, b**) Schematic representation of CBM-deleted (∆CBM) BT4244 (**a**) and HC11 (**b**) mucinases without their BACON/CBM32 or tandem BACON/CBM32 domains, respectively. **c, d**) Mucinase activity assay with different membrane-bound mucin reporters displayed on Tn or T glycoengineered (HEK293-Tn/T) exposed to wild type (WT) and CBM-deleted versions of BT4244 (**c**) and HC11 (**d**) analyzed by flow cytometry. Cells were incubated with mucinases at varying concentrations (0–100 µg/mL) for 1 h. Heat maps show representative cleavage profiles from two independent experiments. Mucinase activity was quantified as the mean fluorescence intensity (MFI) of anti-FLAG tag antibody binding, normalized to the corresponding untreated controls for each mucin reporter-expressing cell line (black indicates high activity, white indicates low activity). **e, f**) Mucinase activity was assessed using an isolated MUC1 reporter produced in HEK293-Tn or HEK293-T cells, monitored by NuPAGE analysis. Mucin reporters (0.5 µg) were incubated with increasing doses of enzyme (0–40 ng) for 1 hour at 37 °C, separated by NuPAGE, and visualized with Krypton fluorescent protein stain. Representative results from two independent experiments are shown.

**
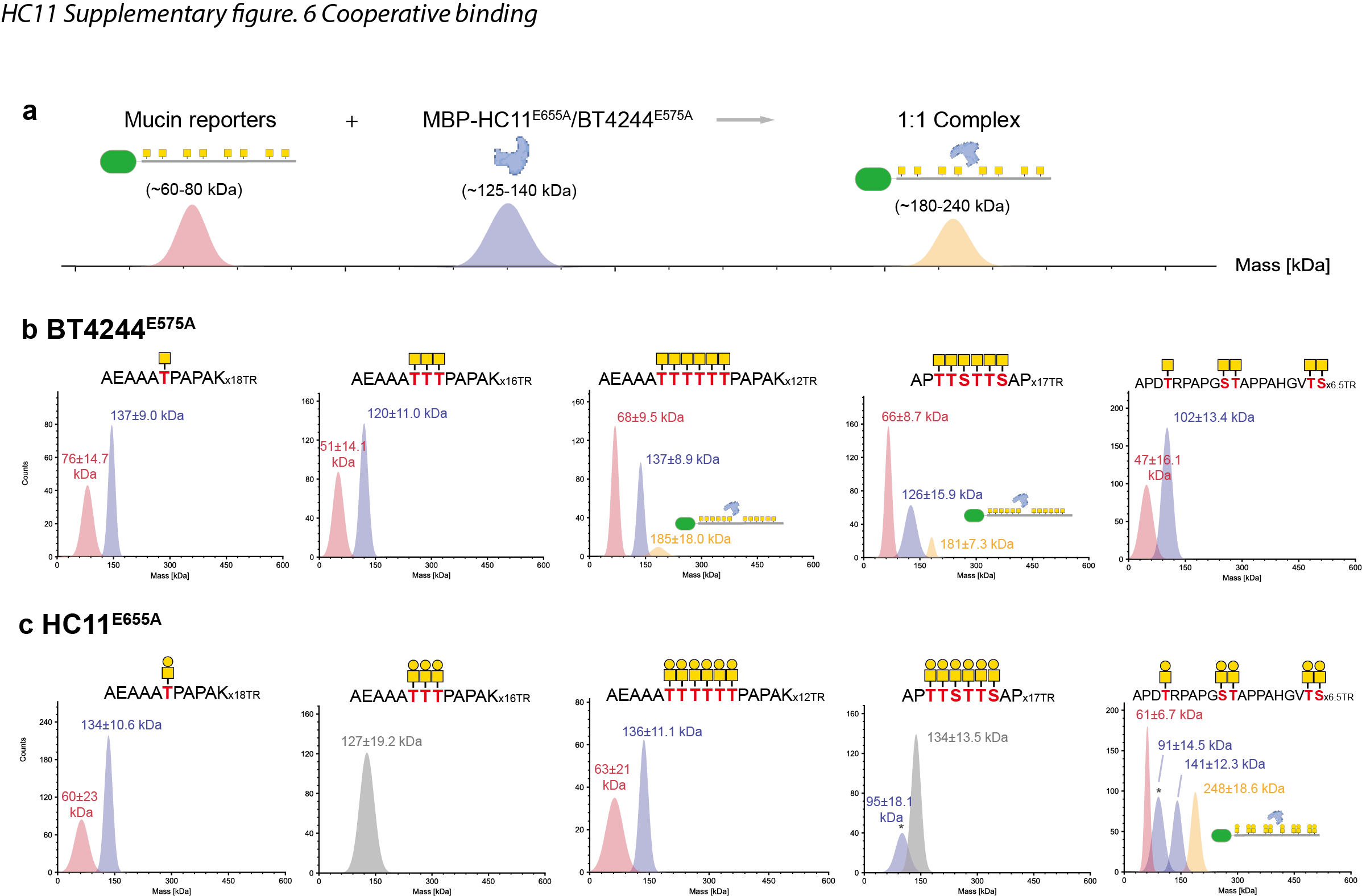
**

**Supplementary Fig. 7. Mass photometry analysis of complex formation between mucin reporters and catalytically inactive BT4244 and HC11 mucinases. a**) Schematic illustration of the mass photometry assay setup with designations used for mucin reporters and catalytically inactive enzymes. **b, c**) Mass photometry histograms showing the binding profile of mucin reporters comprising constructs with 1, 3 and 6 clustered O-glycans, as well as the MUC1 reporter with Tn or T O-glycans incubated with 50 nM of catalytically inactive BT4244 (**b**) or HC11 (**c**). Histograms represent binding interactions observed across at least three independent replicates.


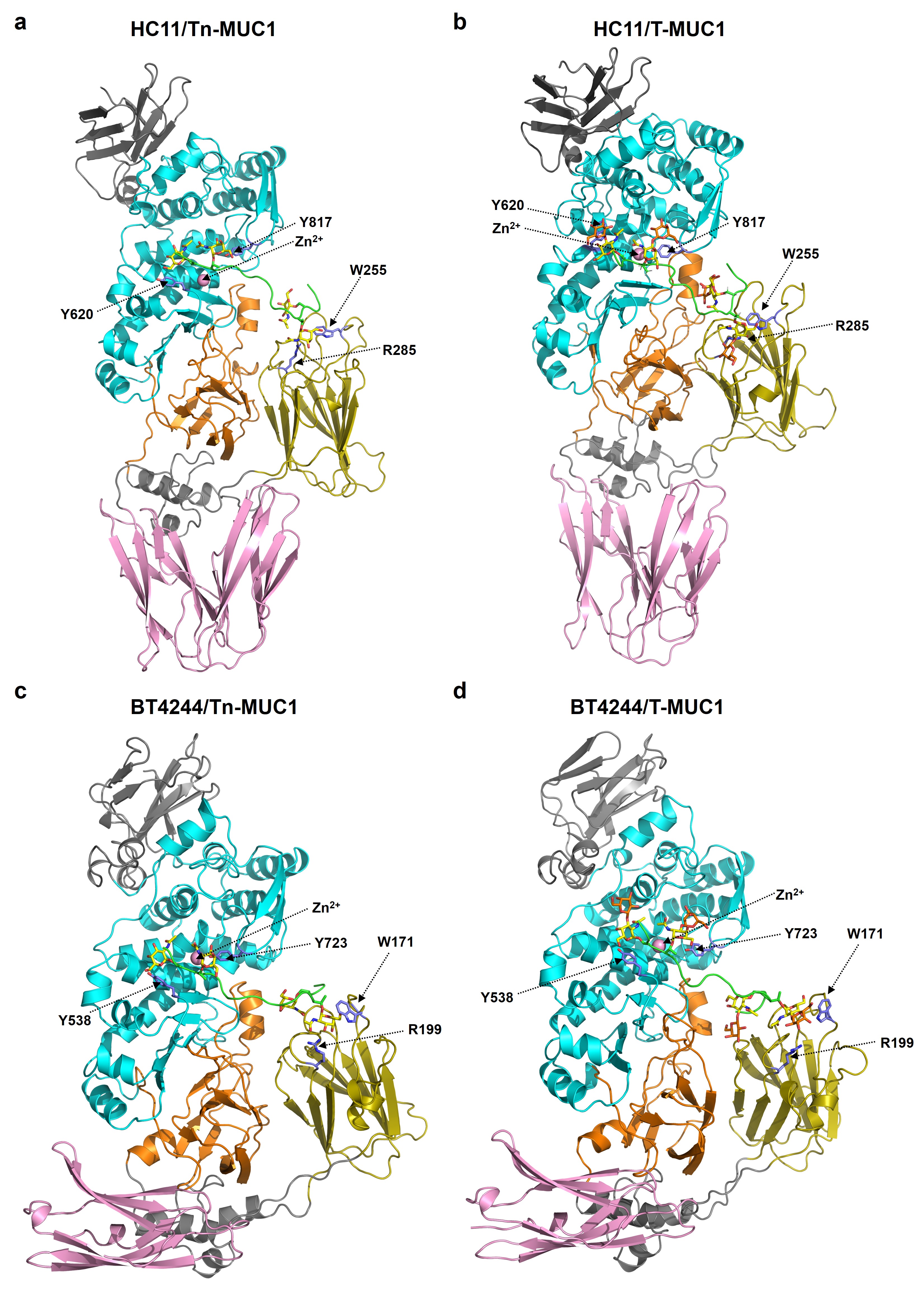


**Supplementary Fig. 8. Representative frames from 300 ns restrained MD simulations of the complex of HC11 and 16-mer Muc1 glycopeptides.** **a**-**d**) MD simulations are shown for HC11 / Tn-Muc1 (**a**), HC11 / T-Muc1 (**b**), BT4244 / Tn-Muc1 (**c**), and BT4244 / T-Muc1 (**d**). The 16-mer Tn and T Muc1 O-glycopeptides were designed from the MUC1 TR (APGS*T*APPAHGVT*S*AP, * indicating Tn/T O-glycans). Carbon atoms of GalNAc and Gal residues are colored yellow and orange, respectively. Key residues that interact with the sugar moieties are shown as violet-colored sticks.

**
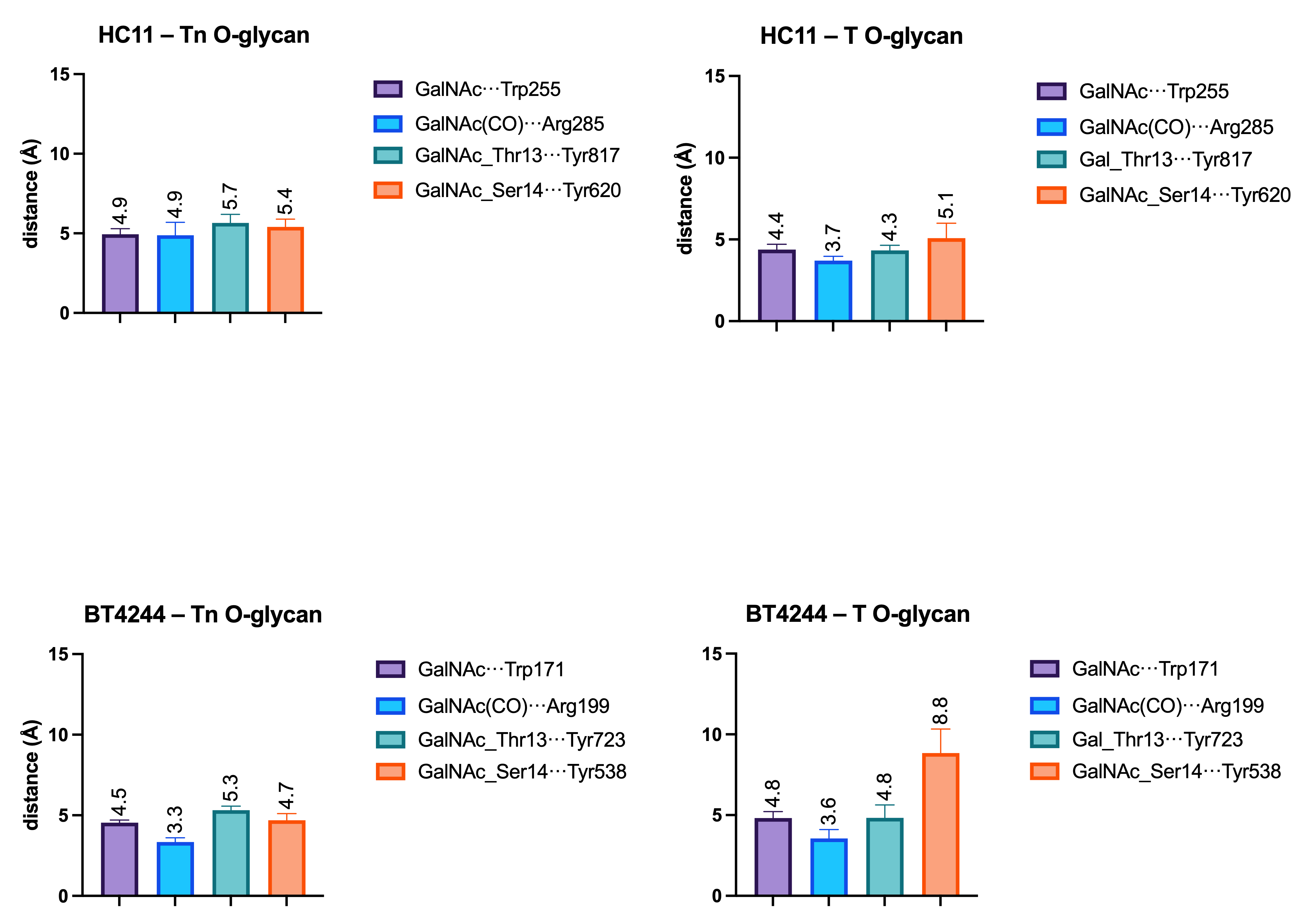
**

**Supplementary Fig. 9. MD simulations for the complexes studied in this work.** Average distances calculated over 300ns were based on the centers of mass of the respective ring systems.

**
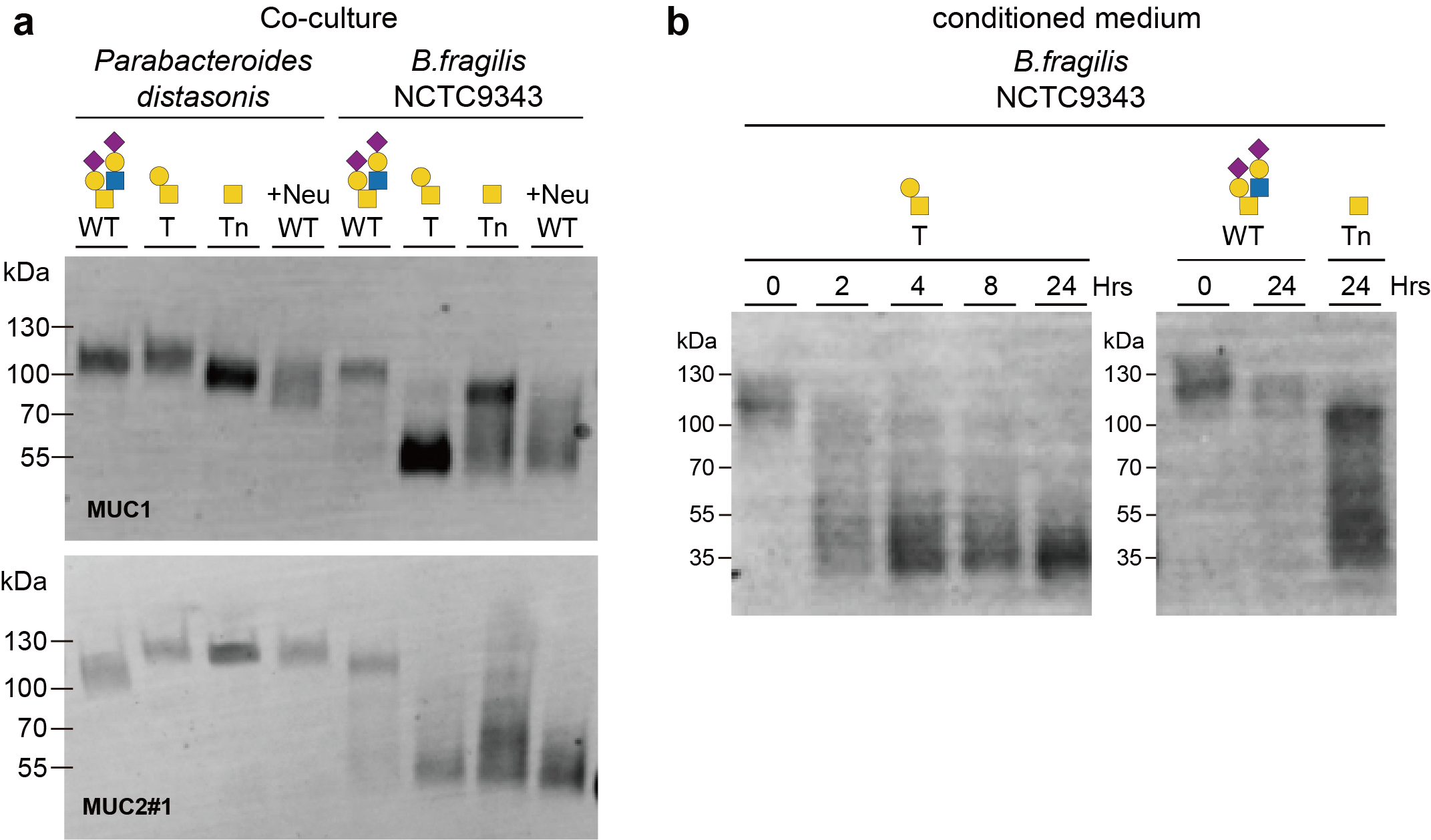
**

**Supplementary Fig. 10. Mucinase activity in anaerobic *B. fragilis* and *P. distasonis* cultures and conditioned medium. a**) Western blots show degradation of mucins reporters co-cultured with *B. fragilis* NCTC 9343 or *P. distasonis* for 18 h in anaerobic cultures. Isolated MUC1 (upper blot) and MUC2 (lower blot) reporters produced in HEK293-WT, HEK293-Tn, or HEK293-T. +Neu indicates addition of recombinant *C. perfringens* sialidase. Culture supernatants were separated by SDS-PAGE gel electrophoresis and blotted on PVDF membranes followed by detection of reporters with anti-FLAG. A representative blot from two independent experiments is shown. **b**) Time-course analysis of conditioned medium from overnight *B. fragilis* NCTC 9343 cultures with MUC2 reporters carrying different O-glycans (T, WT and Tn glycoform). Western blot with anti-FLAG. The experiment was performed twice.

**
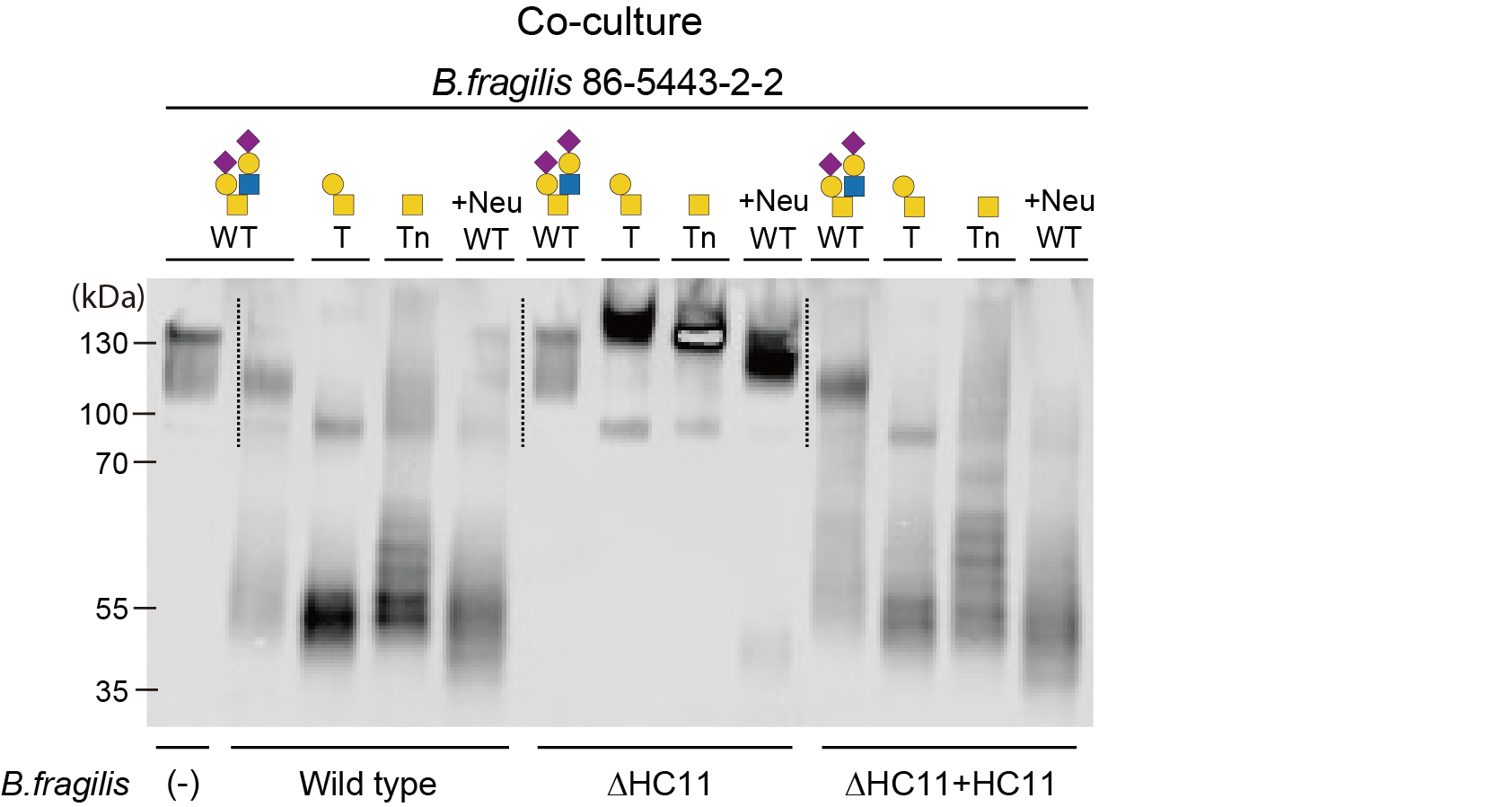
**

**Supplementary Figure 11. Analysis of HC11 T-mucinase activity in culture with *B. fragilis* 86-5443-2-2.** Western blot of degradation of MUC2 reporters with different O-glycans in cultures with wildtype *B. fragilis* 86-5443-2-2, HC11 knockout (ΔHC11), and HC11 rescue (ΔHC11 + HC11) strains after 24 h. +Neu indicates addition of *C. perfringens* sialidase (Neu). Mucin degradation was detected by anti-FLAG. Blot is representative for two independent experiments.
